# Assessing Hospital Resource Utilization with Application to Imaging for Patients Diagnosed with Prostate Cancer

**DOI:** 10.3390/healthcare10020248

**Published:** 2022-01-28

**Authors:** Yazmine Lunn, Rudra Patel, Timothy S. Sokphat, Laura Bourn, Khalil Fields, Anna Fitzgerald, Vandana Sundaresan, Greeshma Thomas, Michael Korvink, Laura H. Gunn

**Affiliations:** 1 School of Data Science, University of North Carolina at Charlotte, Charlotte, NC 28223, USA; ylunn@uncc.edu (Y.L.); rpate182@uncc.edu (R.P.); tsokphat@uncc.edu (T.S.S.); lbourn1@uncc.edu (L.B.); kfield12@uncc.edu (K.F.); afitzg13@uncc.edu (A.F.); vsundare@uncc.edu (V.S.); 2 Department of Public Health Sciences, University of North Carolina at Charlotte, Charlotte, NC 28223, USA; gthoma35@uncc.edu; 3 ITS Data Science, Premier, Inc., Charlotte, NC 28277, USA; michael_korvink@premierinc.com; 4 Faculty of Medicine, School of Public Health, Imperial College London, London W6 8RP, UK

**Keywords:** resource utilization, misutilization, medical imaging, prostate cancer, risk adjustment

## Abstract

Resource utilization measures are typically modeled by relying on clinical characteristics. However, in some settings, those clinical markers are not available, and hospitals are unable to explore potential inefficiencies or resource misutilization. We propose a novel approach to exploring misutilization that solely relies on administrative data in the form of patient characteristics and competing resource utilization, with the latter being a novel addition. We demonstrate this approach in a 2019 patient cohort diagnosed with prostate cancer (*n* = 51,111) across 1056 U.S. healthcare facilities using Premier, Inc.’s (Charlotte, NC, USA) all payor databases. A multivariate logistic regression model was fitted using administrative information and competing resources utilization. A decision curve analysis informed by industry average standards of utilization allows for a definition of misutilization with regards to these industry standards. Odds ratios were extracted at the patient level to demonstrate differences in misutilization by patient characteristics, such as race; Black individuals experienced higher under-utilization compared to White individuals (*p* < 0.0001). Volume-adjusted Poisson rate regression models allow for the identification and ranking of facilities with large departures in utilization. The proposed approach is scalable and easily generalizable to other diseases and resources and can be complemented with clinical information from electronic health record information, when available.

## 1. Introduction

Medical resources may be expensive, in short supply, or have potential negative side effects. Healthcare facilities must consider the frequency with which resources are deployed and identify potential cases of inappropriate utilization, or misutilization. Additionally, the efficient use of resources is a key measure when determining overall healthcare quality. For example, the Yale New Haven Health Services Corporation/Centers for Outcomes Research and Evaluation (CORE) reports that there is an association between resource utilization and efficiency measures as a proponent of their methodology for the Overall Hospital Quality Star Rating, which is a joint methodology with the Centers for Medicare and Medicaid Services (CMS) [1]. Resource utilization also directly impacts financial incentives that acute care facilities receive from the Overall Hospital Quality Star Rating program [1]. Therefore, automated approaches that identify cases of potential resource over-/under-utilization are needed, especially for cases where clinical guidance of optimal utilization is not defined or the inputs needed for assessing utilization are not available (e.g., some patient clinical information). Over-utilization can be defined as the use of a resource that is unlikely to improve a patient’s outcome [2] while under-utilization can be seen as the absence of the use of a resource that would enhance a patient’s outcome [3].

The challenge of achieving homogeneous, optimal resource utilization for all patients across facilities stems from a lack of treatment standardization across professionals and medical entities, as well as the absence of generalized resource utilization models. Such models would both provide a standard for resource utilization to practitioners and also identify potential over-/under-utilization of hospital resources during in-patient hospital stays. When developing models to detect resource over-utilization, researchers have identified patient, clinical, and system-level factors as variables of interest [4]. However, certain clinical factors may not be available for some users, such as those with administrative information and limited access to electronic health records (EHR), rendering some of the available models less generalizable. This also represents an ongoing inequity for hospitals interested in increased utilization efficiency but are unable to collect or use all clinical information needed to utilize such models.

The literature around resource utilization can be coarsely grouped into three types of studies: (1) those that focus on descriptive quantification of resource utilization; (2) studies that focus on the classification of misutilization, oftentimes using disease-specific clinical markers and patient-specific risk assessments; and (3) studies that focus more on the ethics around resource utilization/allocation (e.g., disparities and inequities regarding resource access from a public health perspective or the ethics around decision-making when limited resources are available, such as during the COVID-19 pandemic).

A wide variety of studies have focused on descriptively measuring utilization. Some of these studies rely on self-reported information [5,6,7], while others utilize administrative data from multiple sources (e.g., insurers, facilities, Medicare expenditures, etc.) for resource utilization quantification, oftentimes with a focus on cost assessment [8,9,10,11]. These studies provide coarse descriptions of resource utilization and associated costs at a macro level, but oftentimes do not focus on patient-specific classification or improvements in utilization.

Resource utilization studies oftentimes focus on disease-specific risk/severity assessments and subsequent resource utilization guidelines [12,13]. Clinical diagnoses and EHR provide the information needed to classify patients and identify cases of resource misutilization against predetermined guidelines/classifiers [14,15]. These studies tend to rely more on disease-specific patient information and are less generalizable across diseases and resources.

From a population health standpoint, inequities surrounding access to healthcare resources have been widely covered in the literature and this topic remains a priority of governmental agencies [16,17]. However, general resource scarcity at the overall population level is relatively new in the U.S., though it is more common in lower income countries, and poses new challenges to resource utilization. Resource shortages have been experienced recently across the U.S. with the COVID-19 pandemic, and decisions have had to be made about allocations per patient based on risk evaluations by facility [18], with additional concerns regarding ethical allocation standards and potential liabilities [19,20]. Poor decision-making regarding resource utilization/allocation results in misutilization or untimely utilization [21], and ethical guidelines have since been proposed to allocate resources fairly according to risk assessments and who can benefit most from such resources in times of crises [22,23]; however, the threat of deepening resource access disparities and inequities remains.

Medical imaging, which is the motivating set of resources for this study, has experienced substantial advancements over the last century [24]. As newer techniques and equipment are adopted, advances in medical imaging technology have provided patients with a higher quality of life and extended life expectancies [2]. These advancements have allowed healthcare providers to better treat patients across a wide spectrum of diseases. In turn, they have allowed patients to obtain definitive diagnostic information and, in many cases, bypass the need for costly surgeries and unnecessary invasive procedures [2].

Imaging resources, such as computed tomography (CT) and magnetic resonance imaging (MRI), work independently or in conjunction with one another to provide insights into whether and how to proceed with treatment. In many scenarios, these are alternative, competing resources, and some are more appropriate than others depending on clinical nuances. Incentive programs such as the Merit-based Incentive Payment System (MIPS) under the CMS Quality Payment Program (QPP) include measures designed to reduce resource misutilization by collecting quality metrics from eligible professionals and providing incentive-based pay [25]. Their list of quality measures includes, for example, the avoidance of the overuse of bone scans for staging low-risk prostate cancer patients, the overutilization of imaging studies in melanoma, and appropriate follow-up imaging for incidental thyroid nodules in patients, among others [25].

In the aforementioned Overall Hospital Quality Star Rating by CORE/CMS, the efficient use of medical imaging is a direct component of the scoring criteria [1]. Despite efforts to reduce the inappropriate use of imaging resources through the issuance of guidelines and quality measures across professional societies and policy organizations [26], both the over- and under-utilization of imaging resources occur during in-patient visits. For example, a retrospective cohort study consisting of 9219 men and 30,398 women found high rates of inappropriate imaging among Medicare patients diagnosed with low-risk prostate and breast cancers, respectively [27]. Inappropriate imaging in this instance refers to both over- and under-utilization.

The over-utilization of radiology resources increases overall healthcare expenditure and also exposes patients to unnecessary radiation [2]. For example, the use of bone scans in prostate cancer patients at low risk for metastatic disease is a negative quality indicator within the CMS MIPS program [28]. Additionally, some providers may “upstage” prostate cancer status through the use of a needle biopsy which leads to additional unnecessary imaging for patients who may not meet the clinical criteria [27]. However, the under-utilization of imaging resources can also lead to adverse patient outcomes. For example, an observational study performed between 2004 and 2005 of Medicare patients diagnosed with prostate cancer found that only 60% of men received radiographic staging before treatment to ensure the absence of metastatic disease [28]. This instance of imaging under-utilization demonstrates how the remaining 40% of high-risk prostate cancer patients with potentially undetected metastases underwent inappropriate local therapy with no benefit [28]. While prostate cancer is the second leading cause of cancer-related deaths among American men, early detection and improved treatment options have allowed for more accurate staging and the steady decline in mortality rates in recent years [29]. This creates a high demand for expensive resources such as those related to diagnostic imaging and screening. Patients diagnosed with prostate cancer may also require hospital imaging to determine staging for secondary metastatic disease in lymph nodes, bones, and other organs [30]. The cost of continuing care for prostate cancer was predicted to have the highest increase (at 42%) in medical cost of care from 2010 to 2020 [31]. This would contribute to an overall expenditure acceleration of 124.57 to 157 billion dollars in the U.S. alone during that period [31].

Without access to proper resource utilization models, hospitals could be overlooking cost-saving opportunities and significantly limiting their effectiveness when detecting, localizing, and staging prostate cancer [32]. Depending on the volume and resources available at a facility, hospitals could be over-relying on imaging resources such as bone and CT scans due to their low cost and wide availability [33]. This dependency could overlook certain limitations from using those resources such as the inability to visualize infiltrative (nonscleortic) bone disease or increased examination times [33]. A more expensive alternative that exposes patients to lower radiation, the MRI, has been considered a more clinically robust imaging resource for prostate cancer as it performs similar functions, but has the added benefit of being used as a follow-up tool to detect local and distant recurrence [34]. Understanding how to properly deploy these resources can provide hospitals with the insights necessary to modernize their operations as they pertain to the use of imaging (and other) resources. This could reveal additional benefits such as improvements in defining clinical groups for drug development and enhanced clarity for therapy recommendations [34].

Oftentimes, only administrative information (e.g., patient characteristics, comorbidities, or resources utilized during in-patient visits) is available in lieu of EHR data. In these cases, the identification of over-/under-utilization is not feasible with existing approaches, which rely on multiple clinical markers of patients to identify cancer stages and the corresponding standard of resource utilization. This constraint limits the ability of some healthcare providers to enhance their resource utilization outcomes using existing models. Therefore, novel approaches are needed that do not rely on (but can incorporate, if available) certain patient and clinical characteristics to effectively identify potential resource misutilization. This novel measure of misutilization will be defined against industry standards, since appropriate utilization is not an observable metric without the aforementioned clinical guidelines oftentimes built on EHR. Over-utilization, as defined going forward in this manuscript, will represent actual resource utilization during patient visits in which such utilization would be uncommon under industry standard practices. Conversely, under-utilization will represent a lack of actual resource utilization during patient visits in which the utilization of the resource would have been common under industry standard practices. The threshold to classify commonality will be informed by industry-wide utilization standards. Our aim is to develop a model that assesses variation in resource utilization built on flexible sets of covariates, rather than solely on disease-specific markers. This would allow us to not only identify potential cases of misutilization, but to create a metric of comparison across facilities based on common denominators of available information and to identify facilities which operate substantially different from their peers. Furthermore, this metric could be applied to different diseases/cohorts in an automated way.

Hospital quality performance measures incorporate patient mix into their assessments [35]; however, there is a gap in assessing imaging resource utilization as it pertains to patient characteristics. Our proposed approach aims to fill a gap in the literature by developing a generalized resource utilization framework, demonstrated through assessing the utilization of imaging resources among a prostate cancer cohort. This framework is built on any available clinical and non-clinical patient information, including competing resource utilization, and does not rely on calibrated values from clinical characteristics. We also aim to create a novel metric that identifies under- and over-utilization by facility, again demonstrated within a prostate cancer patient cohort. The proposed approach in this manuscript provides a scalable framework for measuring misutilization and provides facilities with an easy-to-use tool to comprehensively monitor their resource utilization practices against industry-based standard resource utilization practices. This approach is not a replacement for clinical guidelines related to resource utilization, but instead is a forensic analysis of potential misutilization upon the conclusion of patient visits, so that facilities can focus their resource utilization optimization efforts on the areas most likely to depart from industry standards.

## 2. Materials and Methods

### 2.1. Data

De-identified data were extracted from Premier, Inc.’s private all-payor database containing administrative, resource utilization, and financial data [36]. Information regarding 51,111 unique in-patient visits across 1056 facilities with a principal or secondary diagnosis of prostate cancer according to the International Classification of Diseases Tenth Revision (ICD-10) code C61 for malignant neoplasm of the prostate [37] and a discharge date in 2019 were included in this study. Only the first visit in 2019 was included for each patient to avoid over-influence by patients with repeated visits. Data are comprised of three groups of variables: (1) binary response representing the CT scan of the pelvis and abdomen without contrast; (2) patient-level demographic and clinical characteristics, including length of stay (LOS), payor type, ICD class as principal or secondary prostate cancer diagnosis, malignancy history, age group in five year increments, race, source of admission, discharge status, Medicare severity diagnosis-related grouping (MS-DRG), recorded comorbidities defined by the Elixhauser comorbidity methodology [38], and masked facility ID; and (3) competing resources, defined as alternative imaging resources used in prostate cancer monitoring and treatment, which include other CT scans, X-rays, MRIs, and magnetic resonance angiographies (MRAs), ultrasounds, special imaging techniques, nuclear medicine, and miscellaneous diagnostic imaging resources (see Table S1 for imaging variable definitions).

### 2.2. Statistical Analysis

Descriptive statistics were performed across all variables. Low-count standard payor types of charity (*n* = 65; 0.13%) and indigent (*n* = 6; 0.01%) were combined into a single category. Adjacent age groups less than or equal to 45 which contained low counts were collapsed into a single category. Discharge status categories with less than 0.1% of total observations were also combined into a single ‘other’ category. All comorbidities and MS-DRGs with at least 500 observed patient visits (approximately 1% of the sample size) were included as covariates, and as MS-DRGs are grouped at the patient visit level, an additional category was created to group MS-DRGs with fewer than this patient visit count threshold.

Exhaustive enumeration of interactions among competing resources was descriptively explored as an alternative approach to identify unusual resource utilization combinations. To test the statistical differences across the observed combinations of resource usage, a Chi-square test was performed. Network visualizations of tetrachoric correlations were used to visualize pairwise associations. A decision tree was also fitted to explore the associations among competing resource usage and our response utilization.

Three separate multivariate logistic regression models were fitted to explore and compare different sets of explanatory variables: (a) both competing resource and patient-level variables; (b) patient-level variables only; and (c) competing resource variables only. The area under the curve (AUC) was calculated for all three and a test for differences was performed to compare (a) and (b). A logistic link was selected due to the binary nature of the response and the flexibility and interpretability of the coefficients. Additionally, the model outcomes (resource utilization probabilities) can be used in a decision-theoretic classification framework.

Misutilization by patient visit is not observable in most cases without clinical information and guidelines. In order to identify over-/under-utilization without a clinical guideline, a decision curve analysis was performed using commonly available administrative information from model (a) above. This decision curve was fitted and compared to competing model-free approaches of treat all (resource is expected to be used in all patient visits) and treat none (resource is not expected to be used in any patient visit) alternatives. A decision threshold with a positive net benefit was informed by the industry-average standards of resource utilization, so that the results were not affected by thresholds that implied increases or decreases in utilization. Misutilization is, therefore, estimated against industry standards of utilization.

Associations of misutilization and patient characteristics, such as race, were explored and visualized with forest plots and odds ratios and corresponding 95% confidence intervals were calculated.

Histograms were produced to illustrate misclassification rates across facilities, as well as under- and over-utilization rates. Examples of extreme cases of resource misutilization were identified, and corresponding patient visit characteristics were tabulated. To analyze the association between facility volume and misutilization rate, a linear regression analysis was conducted with a log transformation of volume due to skewness in facility volumes. To illustrate the difference in facility volume as it relates to under- and/or over-utilization, a scatterplot was produced against log-volume and distributional differences in misutilization rate by log-volume were demonstrated through a two-sided histogram.

A volume-adjusted Poisson rate regression analysis, built under the assumption of a common misutilization rate per patient visit and hospital, was performed to identify outlying facilities with extreme misclassification rates. Facilities with large departures from the model implied distributional assumptions of homogeneous misutilization rates per patient visit (i.e., higher volume-adjusted rates of mis-, over-, or under-utilization) were identified through 1-sided *p*-values. Bubble plots were created to depict mis-, over-, and under-utilization rates and the corresponding patient volumes for facilities with extreme values. A threshold to identify facilities with extreme values was set at *p* < 0.01. All statistical analyses were conducted in RStudio Cloud version 1.4.

## 3. Results

### 3.1. Descriptive Statistics

Table 1 contains descriptive statistics for all variables. A total of 14,817 admissions (28.99%) were reported to have prostate cancer as their principal diagnosis, and 2775 (5.43%) admissions had a history of malignancy. The mean length of stay was 4.57 days. Most patients were over 65 years (36,982; 72.36%) with the majority of individuals self-identifying as White (36,848; 72.09%), and almost half of the cohort (22,077; 43.19%) were insured by traditional Medicare. Most admissions occurred by physician referral (39,417; 77.12%), and 29,527 (57.77%) of admissions were discharged to home or self-care. The most common MS-DRG (i.e., primary diagnosis grouping for the in-patient visit) was major male pelvic procedures (12,795; 25.03%). Among the 26 observed comorbidities, uncomplicated hypertension was the most common (21,819; 42.69%). A CT scan of the pelvis/abdomen without contrast, the outcome resource, was used in 5990 (11.72%) admissions whereas X-rays were used most widely (28,969; 56.68%) among competing imaging resources, followed by other CT scans (18,630; 36.45%).

### 3.2. Risk Adjusting Resource Utilization

**Figure 1:**
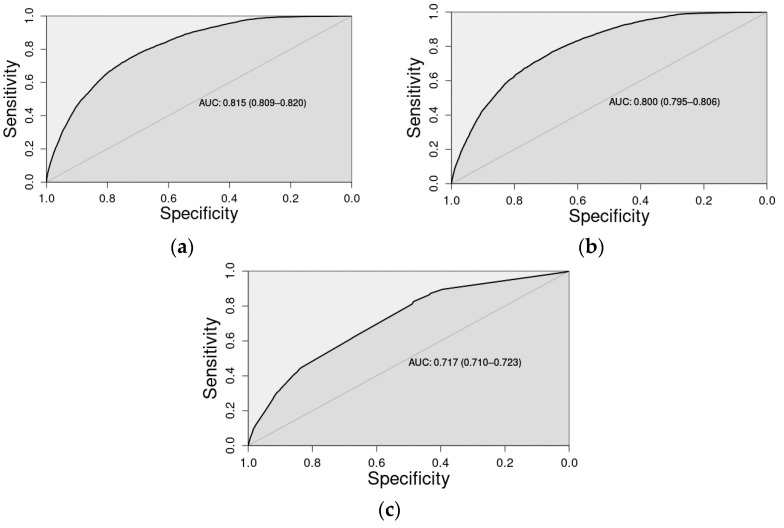
Receiver operating characteristic curves where sensitivity (*y*-axis) is plotted against specificity (*x*-axis), and the area under the curve (AUC) values (corresponding 95% confidence intervals) are provided for the following multivariate logistic regression models: (**a**) patient-level and competing resources information; (**b**) patient-level characteristics only; and (**c**) competing resources only.

Figure 1 represents the area under the (receiver operating characteristic (ROC)) curve (AUC) for the three aforementioned multivariate logistic regression models. Their respective values were: (a) 0.815 (95% CI: 0.809–0.820); (b) 0.800 (95% CI: 0.795–0.806); and (c) 0.717 (95% CI: 0.710–0.723). Competing resources, represented by only seven binary covariates, offered both stand-alone (AUC = 0.717) and marginal explanatory value, as demonstrated through DeLong’s test for AUC differences between the model with patient characteristics and competing resources and the model with only patient characteristics (AUC difference = 0.015; 95% CI: 0.012–0.016; *p* < 0.0001). Table S2 presents the logistic regression analysis results with coefficient estimates, standard errors, and *p*-values for all three models. This highlights that both patient-level and competing resource information can be relevant to explain variability in response resource utilization.

To demonstrate the value of competing resources as an additional set of covariates, they were explored separately. Competing resources clustered in seven variables rendered 128 potential unique combinations of resource usage for each patient visit. However, only 110 unique combinations were observed. Each combination of resources can be represented with seven binary digits, with 1 and 0 representing whether or not, respectively, each of the seven resources was used. For example, among patient visits where none of the competing resources was used (“0000000”), the outcome resource was used in only 3.31% of cases. This indicates that it is relatively rare (and a flag for potential over-utilization) to use only the response resource in isolation, as the proportion of utilization among those who also used some competing resource was markedly different (16.15%). This is summarized in Table S3. A chi-squared test for differences in outcome utilization across the 110 observed combinations of competing resources rendered a statistic of 4411.5 (*p* < 0.0001). Information regarding the probability of joint utilization can also be visually extracted through network sub-analyses of tetrachoric correlations (Figures S1 and S2) or through a decision tree linking joint alternative resource utilization with outcome utilization (Figure S3). The marginal explanatory power of competing resource interactions was found to be minimal compared to the model which solely included the main effects (AUC difference of 0.009) and relative to the large loss in degrees of freedom, so they were not included in the final model.

### 3.3. Misutilization

Building on the fitted probabilities from the multivariate logistic model with patient-level characteristics and competing resources, Table S4 outlines examples of patient visits with extreme probabilities of response resource utilization where the actual resource utilization was not aligned. The top five patient visits listed had low predicted probabilities of using the response resource, but the resource was used in practice. The bottom five patient visits listed had high predicted probabilities of using the response resource, but the resource was not used. While these cases represent extreme examples, in order to define potential misutilization for *all* patient visits, a decision threshold for the fitted probabilities is needed.

**Figure 2:**
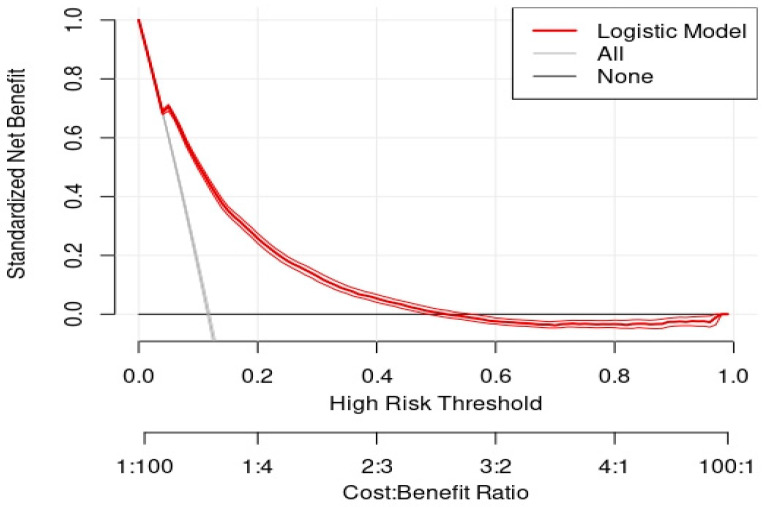
Decision curve analysis across possible probability threshold values for resource utilization using the fitted probabilities from the multivariate logistic regression model with patient-level characteristics and competing resources. Comparative alternative approaches are treat all (outcome resource is expected to be used in all patient visits) and treat none (outcome resource is not expected to be used in any patient visit) alternatives. Net benefit (*y*-axis) is displayed by threshold and cost-benefit ratio (*x*-axis).

Figure 2 shows a decision curve analysis across possible probability threshold values for resource utilization. This decision curve shows a positive net benefit for threshold probability values between 0 and 0.48. This proposed model adds value identifying utilization over treat all (assume utilization by all) and treat none (assume utilization by none) strategies in the range between probability thresholds of 0.05 and 0.48. When determining the decision threshold, we considered a value that mirrored a similar intensity of utilization of the response resource, which was used in 11.72% of patient visits. By selecting the decision threshold of 0.20 (20% probability of outcome resource utilization), we achieved a similar resource utilization of 12.77%, as already achieved in practice across hospitals. Using this threshold, we identified a 17.48% misutilization rate, comprised of 68.85% over-utilization and 31.15% under-utilization. The aforementioned threshold corresponds to a 1:4 cost-benefit tradeoff, where cost relates to under-utilization (clinical net health cost).

Table S5 lists the top 10 facilities (among those with at least 30 patient visits) with the lowest and highest levels of mis-, over-, and under-utilization of the response resource. Facility 1221, with 30 cohort patients, was estimated to have 46.67% misutilization and 30% over-utilization whereas facilities 321, 456, and 466 reported 0% mis- and under-utilization for 32, 49, and 55 patients, respectively.

**Figure 3:**
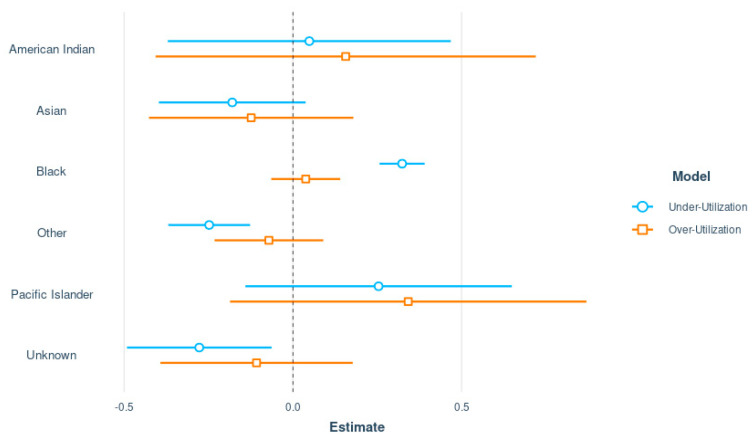
Forest plot showing the logistic regression coefficient estimates representing the associations between race and under- and over-utilization, with White patients representing the reference category.

Table 2 shows a significant positive association of under-utilization among Black participants when compared to White participants (odds ratio = 1.38; 95% CI 1.29–1.48; *p* < 0.0001), and the reverse was observed among those identified as other (OR = 0.78; 95% CI 0.69–0.88; *p* < 0.0001) and unknown (OR = 0.76; 95% CI 0.61–0.93; *p* = 0.0112) race. These differences by race are also depicted in the forest plot in Figure 3. No significant differences were observed for over-utilization of the response resource by race. Tables S6–S11 provide additional examples of associations between misutilization and ICD class and age groups, as well as the associations of under- (over-) utilization with payor type.

**Figure 4:**
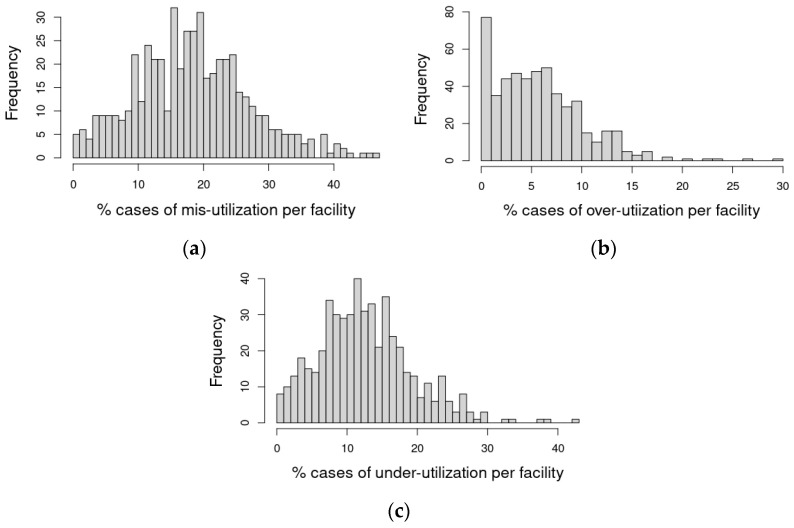
Histograms portraying the observed distribution of (**a**) mis-, (**b**) over-, and (**c**) under-utilization rates by facility using the multivariate logistic model (containing both patient and competing resources information) fitted probabilities and a probability threshold for utilization of 20%.

Figure 4 shows the distribution of potential mis-, over-, and under-utilization rates of the response resource by facility. The distribution of the percentage of patient visits per facility with identified misutilization is more symmetric, as seen in panel (a). Over-utilization, represented in panel (b), shows that for most facilities, less than approximately 20% of patient visits utilized the response resource although they were not predicted to use it. Panel (c), representing under-utilization, shows that in most facilities, less than approximately 30% of patient visits per facility did not use the response resource, although they were predicted to use it. These figures are influenced by the different volumes per facility; hence, we provide a volume-adjusted metric to follow.

In order to identify facilities with potentially larger misutilization rates, Poisson rate regression models for the mis-/over-/under-utilization counts by facility, adjusted by volume and with constant underlying rates, revealed that out of the total 1056 facilities, 66, 79, and 55 facilities departed substantially from the theoretical distribution of common misutilization rates, with one-sided right-side *p*-values < 0.01 for misutilization, over-utilization, and under-utilization, respectively. The outcomes of these models allow for both the identification of hospitals more likely to utilize resources in a non-standard form versus industry standards, and also the ranking of hospitals by mis-, over-, and under-utilization rate while adjusting for volume differences.

**Figure 5:**
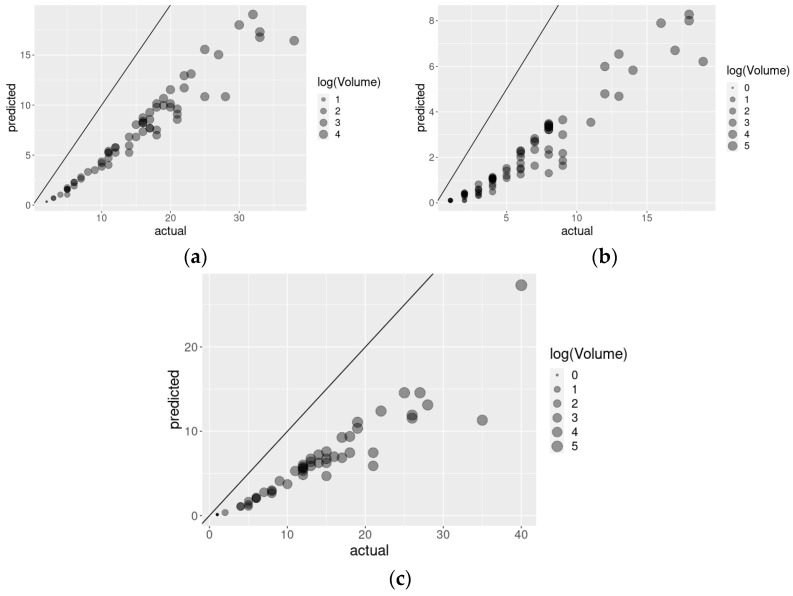
Bubble plots where predicted (**a**) mis-, (**b**) over-, and (**c**) under-utilization counts (*y*-axis) are plotted against the actual values (*x*-axis) from the Poisson rate regression models where each bubble represents the facilities for which the *p*-values are below 0.01 and with the size of each bubble representing the log volume of patients in the facility.

Figure 5 portrays the aforementioned facilities with *p* < 0.01, with the diagonal line representing the misutilization rates aligned with model expectations, the axes representing actual (*x*-axis) and predicted (*y*-axis) rates, and the bubble sizes representing log volumes. Values below the diagonal lines represent facilities with higher-than-predicted levels of mis-/over-/under-utilization. The hospital with the largest volume among those identified with large misutilization rates had only 109 patients, while 11.74% of hospitals had larger volumes and were not flagged (e.g., none of the hospitals in the largest decile by volume were identified in the group of most likely misutilization). The hospital that was identified to be over-utilizing the response resource the most had 152 patients, with only 5.87% of hospitals experiencing larger volumes. Finally, the hospital identified to be under-utilizing the response resource the most had 227 patients, while only 1.61% of hospitals had larger volumes than that.

**Figure 6:**
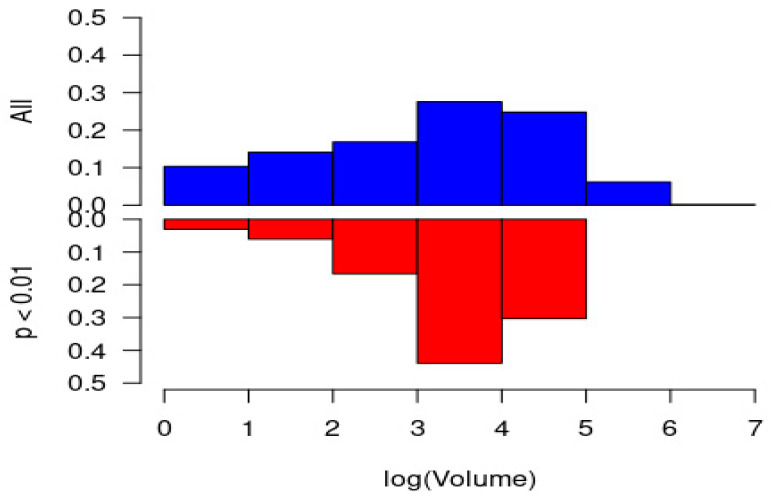
Distribution of cohort patient volume (log scale) for all hospitals (top, blue) versus those with substantial levels of misutilization, identified as *p* < 0.01 (bottom, red).

Figure 6 shows the distribution of all hospitals’ log(volume) (top, blue) against the distribution of log(volume) for hospitals identified as having misutilized the response resource the most (red, bottom). The plot shows how mid-size hospitals were identified to have misutilized the response resources the most.

**Figure 7:**
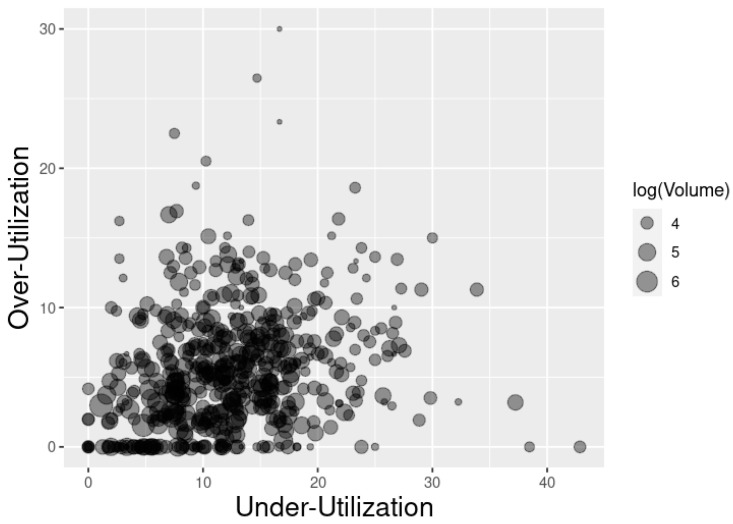
Bubble plot of under- and over-utilization with log(volume) representing facility size.

Figure 7 portrays under- and over-utilization by facility, with the bubble size representing facility (log)volume. Simple (independent) linear regressions to assess the associations of log(volume) with over- and under-utilization show strong negative associations (regression coefficients of −2.00 and −1.49, respectively, and *p* < 0.0001 for both regressions).

## 4. Discussion

Facilities and other stakeholders cannot easily explore (and stay abreast of) time-evolving and oftentimes unavailable clinical guidelines of utilization by resource and disease and analyze each such pair against these clinical guidelines to measure their level of resource misutilization. Such an approach would require thousands of these clinical guidelines (one per combination of resource and disease) and corresponding metrics, and most facilities will not have the resources, access to data, and/or expertise for such level of micro-monitoring. Therefore, a more generalizable, universally available, and fully scalable methodology is needed to address the wider problem of identifying potential areas of resource misutilization at the facility level across diseases and resources.

When clinical information is not available, predicted resource usage calculated from administrative data (both patient-level characteristics and competing resources information) can be used to determine misutilization by combining: (1) a predictive model for utilization at the patient visit level; (2) a decision-analytic approach grounded in empirical utilization levels; and (3) a volume-adjusted Poisson rate regression metric at the facility level. This approach is applicable to a wide variety of health conditions and hospital resources, since it does not rely on disease-specific clinical markers, and it can be of particular benefit for hospitals with limited tools available to self-assess and optimize their resource utilization practices.

Our results for the motivating example of prostate cancer and CT scans demonstrate that misutilization rates by facility are negatively correlated with patient volume. There could be a variety of factors that could explain why larger hospitals (those with prostate cancer patient volumes over approximately 100) experience lower levels of misutilization. One possible explanation could be that larger facilities may have more established policies and higher accountability for appropriately utilizing resources, which may be in higher demand in larger hospitals. Moreover, larger hospitals may align more with consistent/more widely accepted industry-based practices, while smaller facilities may experience larger heterogeneity in their clinical approach to imaging utilization. The proposed Poisson rate regression approach provides volume-adjusted metrics that allow for inter-facility comparisons of potential misutilization. This facility-specific metric allows facilities with extreme levels of misutilization against industry practices to investigate their internal resource utilization practices and adjust them, if needed, to match any existing clinical guidelines or standard industry practices.

We found that mid-sized hospitals (those with prostate cancer patient volumes around 20–100) contributed in larger amounts to the identified cases of misutilization. Ullrich et al. [39] recently compared CT utilization in a single emergency department (ED) on high against low volume days over a five-year period and found that increased ED volume does not significantly change the rate in which physicians utilize CT scans for patients with abdomen pain [39]. However, their study focused on intra-facility variability, rather than inter-facility differences in misutilization by volume.

The AUC estimates indicated a good fit when using a multivariate logistic model containing patient characteristics and competing resource information, the latter of which is a novel set of information considered in this study. Competing resources demonstrated their value both in stand-alone form (e.g., even if patient characteristics were not available) and also in combination with patient-level information.

Higher rates of under-utilization versus over-utilization were identified in this study, indicating there were more instances of patients not receiving a CT scan of the pelvis/abdomen without contrast when it would be expected to be used based on industry-wide standards. CT scans are often used for determining prostate cancer volume or for high-risk patients [30]. These results indicate that physicians may be using alternative (or no) imaging methods when a CT scan might be better suited for the imaging purpose, such as determining specific treatments based on tumor volume. Under-utilization rates may be further fueled by decision protocols put in place by hospitals and resource availability.

When considering the role patient characteristics play in resource misutilization, we found significant misutilization by age among patients older than 70 (see Table S9), which aligns with the findings in [40] regarding higher rates of resource misutilization for elderly patients. They found statistically significant evidence that as patients aged, they had lower odds of receiving multi-parametric MRIs (mpMRIs) (*p* ≤ 0.01 for all risk stratified multivariate analyses) [40]. Although MRIs may be more desirable, in general, than CT scans without contrast when staging because of their exceptional soft tissue resolution, CT scans are more beneficial when evaluating advanced disease with adjacent organ invasion and distant lymphadenopathy [41]. Note that these results are specific to the combination of disease and resource within our motivating example and cannot be extrapolated to these resources for other diseases/cohorts. The proposed approach is fully data-driven and allows for heterogeneity in associations by disease and resource.

A prior study showed that low risk, non-private payors experienced lower odds of using mpMRI versus low-risk private payors (*p* = 0.02) [40]. We found that all private payors (commercial indemnity, direct employer contract, managed care capitated, managed care non-capitated, workers compensation, other) and some non-private payors (Medicaid managed care non-capitated, Medicare managed care capitated, and other government payors) experienced significant negative associations with misutilization, indicating that there is less under- and/or over-utilization among these private and non-private payors compared to patients with traditional Medicare (*p* ≤ 0.05) (see Table S11).

We found that there was no significant over-utilization by race when comparing Black with White participants. However, we found strong evidence of greater under-utilization among Black compared to White patients (odds ratio (OR) 1.38; 95% CI 1.29–1.48). This aligns with Ajayi et al. [40], who reported significantly lower odds of mpMRI utilization among low-risk Black vs. White participants (OR 0.21; 95% CI 0.08–0.55). Differences in misutilization rates by patient characteristics such as age, payor type, or race demonstrate the need for further research into equity-related resource utilization disparities and the root/latent causes behind them.

### Strengths and Limitations

Under- and over-utilization are defined based on a general practice format rather than in clinical appropriateness, which would need access to patients’ clinical markers for the disease, such as the Gleason scale. Thus, utilization rates might be imprecise because the dataset does not contain information specific to PSA levels, Gleason scores, or tumor stage, all of which play a role in the risk-level of the patient and the ultimate use of a CT scan [30]. The Gleason scale, for example, is a tool used to determine prostate cancer severity, which can be beneficial in determining which imaging resource should be used [42]. The lack of disease-specific clinical markers is both a strength of this study, as it allows for extrapolability and scalability across diseases and resources with limited administrative information, but it is also a limitation, as clinical markers could arguably provide more information about resource utilization appropriateness. Most studies on utilization rely on EHR data and the availability of these clinical markers, deterring any analysis when only non-clinical administrative data is available. Such clinical markers can be easily incorporated as part of the covariate set in our proposed approach, when available. However, they are not a prerequisite in our approach for extracting a well-calibrated measure of misutilization against industry standards. We provide an alternative where clinical markers are useful if available, yet not necessary, to explore potential misutilization at both the patient visit level and at the facility level. Moreover, from a modeling perspective, alternative classification approaches are possible and could be explored in future research. However, our approach can handle the unsupervised nature of the problem, where appropriate utilization by patient visit is not an observable metric.

Administrative information is limited and provides a partial snapshot of patient characteristics. Latent factors unavailable in administrative datasets may influence some of the associations described in the Results section. For example, it is unclear whether race would remain a statistically significant factor (or to what extent) upon accounting for other (correlated) variables such as economic status or poverty, which were not available. Moreover, the associations highlighted in the Results section are not meant to indicate causality, but to demonstrate differences in utilization and misutilization by coarsely defined patient characteristics.

The lack of complete patient information does not invalidate the proposed approach, which is to measure utilization standards and define a misutilization metric upon accounting for all available and relevant patient-level information. This set of information will always be incomplete, but the approach is meant to provide facilities and other stakeholders with a tool that offers practical, scalable solutions with partial information (i.e., limited or no clinical information, and partial non-clinical information).

Facilities with larger volumes and variety of resources at their disposal could potentially have a greater influence in defining what is commonly performed in practice and what our model identifies as misutilization of resources. However, this is mitigated by the large number of small-to-mid size facilities, which comprise a large number of patients in our cohort.

This study utilized a variety of administrative information, including the novelty of inclusion of competing resource information, which has been demonstrated to be beneficial in explaining the variability in resource utilization. This is a practical approach that allows hospital systems which do not have access to the necessary information for clinically driven benchmarking of misutilization to have a tool at their disposal to explore and address potential cases of misutilization. Another strength of this study is the possibility to identify and rank facilities based on evidence of deviations in utilization patterns when compared to industry standards. This approach relies on the assumption that industry averages are appropriate targets, whereas a clinical definition of misutilization, when available, would be a more appropriate benchmark for classifying ‘misutilization’. However, this assumption can be easily relaxed by selecting a set of industry leading healthcare facilities and fitting the multivariate logistic model with administrative covariates for these facilities only and using it to estimate misutilization among other facilities. Then, any misutilization could be defined against the standards of practice of such industry leaders.

Importantly, note that we do not intend for this model to provide or replace clinical decision-making guidelines, nor any patient-level rules for resource utilization during the patient’s stay. Our approach is forensic in nature and intends to provide a metric for computing the likelihood of misutilization across patient visits for facilities after patient visits are concluded, so that facilities (and other stakeholders) can investigate whether and why their resource utilization practices are different from others in the industry. By using our approach, facilities can be flagged for risks of misutilization across all pairs of diseases and resources based on their history of utilization across patient visits and focus their resource utilization optimization efforts on the pairs that are more likely to depart from industry standards.

Finally, this approach is disease- and resource-blind. When exploring misutilization by facility across a wide range of diseases and resources, there will be an incredibly large number of such analyses that would need to be performed. Our proposed pragmatic approach provides an automated way to explore misutilization by facility across these dimensions without the need for clinical markers or knowledge of/adaptation to constantly changing disease-specific clinical guidelines. If implemented across resources and diseases, this method would provide facilities and other stakeholders with a comprehensive picture of their resource utilization practices and the disease-resource pairs where they diverge the most from industry standards.

## 5. Conclusions

Assessment of resource utilization across diseases and resources is a complex problem in need of generalizable and scalable models. Model calibration based on clinical patient characteristics and disease-specific markers is oftentimes not plausible, and such clinical information is sometimes unavailable to those responsible for identifying potential resource misutilization. The approach proposed in this study, which can be generalized across diseases and resources, provides a metric to identify potential misutilization against industry standards, which is demonstrated through the measurement of under- and over-utilization of a hospital imaging resource during in-patient hospital stays among patients diagnosed with prostate cancer. We found that the under- and over-utilization of CT scans of the pelvis and abdomen without contrast were significantly lower among hospitals with larger patient volumes. This study provides an example of a novel, data-driven approach using administrative/practice-based information, though clinical information can be incorporated when available. This is especially useful for facilities that lack the tools or funding to perform detailed analysis across all their resource utilization practices. Such facilities and other stakeholders would benefit from understanding which disease–resource pairs are most likely to benefit from the optimization of resource utilization practices or alignment with industry standards or benchmarks. Future research includes scaling this approach across patient cohorts and hospital resources, and the joint modelling of interchangeable resources.

## Figures and Tables

**Table 1:** Descriptive statistics (counts/means and percentages/standard deviations) of patient characteristics, competing resources, and the outcome resource.

Characteristics	Count/Mean (%/SD)
Length of Stay (days)	4.57 (5.44)
Cost-type: Procedural	30,559 (59.79)
Prostate Cancer as Principal ICD-10 Classification	14,817 (28.99)
Age (years)	
≤45	170 (0.33)
46–50	597 (1.17)
51–55	1977 (3.87)
56–60	4408 (8.62)
61–65	6977 (13.65)
66–70	8234 (16.11)
71–75	8168 (15.98)
76–80	7305 (14.29)
81–85	6155 (12.04)
>85	7120 (13.93)
Race	
American Indian	206 (0.40)
Asian	929 (1.82)
Black	8572 (16.77)
Pacific Islander	200 (0.39)
Unknown	1037 (2.03)
White	36,848 (72.09)
Other	3319 (6.49)
Payor type	
Charity or Indigent	71 (0.14)
Commercial Indemnity	2706 (5.29)
Direct Employer Contract	96 (0.19)
Managed Care Capitated	168 (0.33)
Managed Care Non-Capitated	8223 (16.09)
Medicaid Managed Care Capitated	264 (0.52)
Medicaid Managed Care Non-Capitated	1209 (2.37)
Medicaid Traditional	716 (1.40)
Medicare Managed Care Capitated	3373 (6.60)
Medicare Managed Care Non-Capitated	9615 (18.81)
Medicare Traditional	22,077 (43.19)
Other Government Payors	1245 (2.44)
Self Pay	439 (0.86)
Workers Compensation	63 (0.12)
Other	846 (1.66)
Point of Origin ^1^	
Clinic	7255 (14.19)
Court/Law Enforcement	38 (0.07)
Information Not Available	431 (0.84)
Non-Healthcare Facility (Physician Referral)	39,417 (77.12)
Transfer from a Hospital (Different Facility)	2607 (5.10)
Transfer from SNF ^2^ or ICF ^3^	675 (1.32)
Transfer from Ambulatory Surgical Center	51 (0.10)
Transfer from Another Healthcare Facility	427 (0.84)
Transfer from Hospice and is Under a Hospice Plan of Care or Enrolled in a Hospice Program	15 (0.03)
Transfer from Hospital Inpatient in the Same Facility Resulting in a Separate Claim to the Payor	195 (0.38)
Discharge Status	
Court/Law Enforcement	65 (0.13)
Expired	1673 (3.27)
Home Health Organization	7732 (15.13)
Home or Self Care	29,527 (57.77)
Hospice Home	1414 (2.77)
Hospice Medical Facility	1100 (2.15)
Left Against Medical Advice	241 (0.47)
Transferred to a Long-Term Care Hospital	235 (0.46)
Transferred to Another Rehabilitation Facility	1273 (2.49)
Transferred to ICF ^2^	185 (0.36)
Transferred to Other Facility	712 (1.39)
Transferred to SNF ^3^	6478 (12.67)
Transferred to Swing Bed	124 (0.24)
Other	352 (0.69)
Medicare Severity Diagnosis Related Groups (MS-DRGs)	
Acute Myocardial Infarction (Discharged Alive)	575 (1.13)
Cardiac Arrhythmia Conduction Disorders	732 (1.43)
Esophagitis, Gastroenteritis and Miscellaneous Digestive Disorders	558 (1.09)
Gastrointestinal Hemorrhage	937 (1.83)
Heart Failure (Shock)	1375 (2.69)
Infectious Parasitic Diseases with Operating Room Procedure	560 (1.10)
Intracranial Hemorrhage or Cerebral Infarction	775 (1.52)
Kidney/Urinary Tract Infections	891 (1.74)
Major Joint Replacement or Reattachment of Lower Extremity	758 (1.48)
Major Male Pelvic Procedures	12,795 (25.03)
Malignancy of Male Reproductive System	1104 (2.16)
Miscellaneous Disorders of Nutrition Metabolism Fluids Electrolytes	720 (1.41)
Other Kidney/Urinary Tract Diagnoses	1521 (2.98)
Pathological Fractures Musculoskeletal Connective Tissue Malignancy	832 (1.63)
Percutaneous Cardiovascular Procedure with Stent	594 (1.16)
Renal Failure	1514 (2.96)
Septicemia or Severe Sepsis without Mechanical Ventilation > 96 h	3749 (7.34)
Simple Pneumonia Pleurisy	944 (1.85)
Other	20,177 (39.48)
Comorbidities	
Alcohol Abuse	1672 (3.27)
Anemia Deficiency	2394 (4.68)
Cardiac Arrhythmia	14,830 (29.02)
Chronic Pulmonary Disease	9784 (19.14)
Coagulopathy	4546 (8.89)
Congestive Heart Failure	9915 (19.40)
Depression	4496 (8.80)
Diabetes-Complicated	8258 (16.16)
Diabetes-Uncomplicated	6411 (12.54)
Drug Abuse	1007 (1.97)
Fluid and Electrolyte Disorders	17,098 (33.45)
Hypertension-Complicated	14,584 (28.53)
Hypertension-Uncomplicated	21,819 (42.69)
Hypothyroidism	4503 (8.81)
Liver Disease	2051 (4.01)
Lymphoma	673 (1.32)
Metastatic Cancer	15,257 (29.85)
Obesity	6232 (12.19)
Other Neurological Disorders	6071 (11.88)
Paralysis	1135 (2.22)
Peripheral Vascular Disorders	4740 (9.27)
Pulmonary Circulation Disorders	2612 (5.11)
Renal Failure	12,320 (24.10)
Rheumatoid Arthritis Collagen	887 (1.74)
Valvular Disease	4235 (8.29)
Weight Loss	5459 (10.68)
Competing Imaging Resources	
CT Scans (Excluding Outcome Resource)	18,630 (36.45)
MRIs and MRAs ^4^	5158 (10.09)
Miscellaneous Imaging	841 (1.65)
Nuclear Medicine	2826 (5.53)
Special Imaging Techniques—All Imaging	3384 (6.62)
Ultrasounds	6493 (12.70)
X-rays	28,969 (56.68)
Outcome Imaging Resource	
CT Scan of Pelvis/Abdomen without Contrast	5990 (11.72)

^1^ Point of Origin: Patient’s source of admission. ^2^ SNF: Skilled Nursing Facility. ^3^ ICF: Intermediate Care Facility. ^4^ MRI and MRA: Magnetic Resonance Imaging and Magnetic Resonance Angiography.

**Table 2:** Odds ratios for associations between race and over- and under-utilization of the response resource, with White patients representing the reference category.

Race	Under-Utilization			Over-Utilization		
	OR	95% CI	*p*	OR	95% CI	*p*
American Indian	1.05	0.67–1.57	0.8200	1.17	0.63–1.97	0.5864
Asian	0.84	0.67–1.03	0.1055	0.88	0.64–1.18	0.4236
Black	1.38	1.29–1.48	<0.0001	1.04	0.94–1.15	0.4648
Pacific Islander	1.29	0.85–1.88	0.2075	1.41	0.80–2.30	0.2045
Unknown	0.76	0.61–0.93	0.0112	0.90	0.67–1.18	0.4590
Other	0.78	0.69–0.88	<0.0001	0.93	0.79–1.09	0.3872

## Data Availability

Data is owned by Premier, Inc. and is available upon agreement with their Data Management services.

## Supplementary Materials

The following supporting information can be downloaded at: https://www.mdpi.com/article/10.3390/healthcare10020248/s1, Table S1: Definitions of competing resource variables; Table S2: Multivariate logistic regression analysis results with coefficients (Coef), standard errors (SE), and *p*-values (*p*) for the three models: (a) patient characteristics and competing resource utilization; (b) patient characteristics only; and (c) competing resource utilization only; Table S3: Binary representation of competing resources indicating the usage of different combinations of resources (0 = not used; 1 = used) along with the count of cases where the outcome resource is used and not used; Table S4: Patient characteristics for the top and bottom five patient visits where predicted resource usage probabilities and actual resource utilization are not aligned; Table S5: Examples of the top and bottom 5 facilities, among those with at least 30 patients, based on the percentage of mis- (left), over- (middle), and under- (right) utilization of resources along with the count of patients in each facility receiving the outcome imaging resource; Table S6: Logistic regression results for the association between misutilization by patient-visit (binary) and ICD class; Table S7: Odds ratios, corresponding 95% CIs, and *p*-values for the association between misutilization and ICD class; Table S8: Logistic regression results for the association between misutilization and age groups; Table S9: Odds ratios, corresponding 95% CIs, and *p*-values for the association between misutilization and age groups; Table S10: Logistic regression results for the associations between over- and under-utilization with payor type; Table S11: Odds ratios, corresponding 95% CIs, and *p*-values for the associations between over- and under-utilization with payor type; Figure S1: Network analysis for associations (using tetrachoric correlations) among all competing resources and the response resource; Figure S2: Network analysis for associations (using tetrachoric correlations) of resources for patient visits with the utilization of at least one competing resource; Figure S3: Decision tree analysis to isolate different patterns in resource utilization by demonstrating the value of information of competing resources with the response resource.
