# Supplementary material for: Assessing Hospital Resource Utilization with Application to Imaging for Patients Diagnosed with Prostate Cancer
Source: Healthcare (Basel). 2022 Jan 28;10(2):248. doi: 10.3390/healthcare10020248 (PMC8872431; doi:10.3390/healthcare10020248)
Supplement: Supplementary file 1 [file healthcare-10-00248-s001.zip › healthcare-1547378-supplementary.pdf]

SUPPLEMENTARY MATERIALS

Content	Page(s)
Table S1. Definitions of competing resource variables.	2-12
Table S2. Multivariate logistic regression analysis results with coefficients (Coef), standard errors (SE), and p-values (p) for the three models: (a) patient characteristics and competing resource utilization; (b) patient characteristics only; and (c) competing resource utilization only.	13-16
Table S3. Binary representation of competing resources indicating the usage of different combinations of resources (0=not used; 1=used) along with the count of cases where the outcome resource is used and not used.	17-19
Table S4. Patient characteristics for the top and bottom five patient visits where predicted resource usage probabilities and actual resource utilization are not aligned.	20-23
Table S5. Examples of the top and bottom 5 facilities, among those with at least 30 patients, based on the percentage of mis- (left), over- (middle), and under- (right) utilization of resources along with the count of patients in each facility receiving the outcome imaging resource.	24
Table S6. Logistic regression results for the association between misutilization by patient-visit (binary) and ICD class.	25
Table S7. Odds ratios, corresponding 95% CIs, and p-values for the association between misutilization and ICD class.	26
Table S8. Logistic regression results for the association between misutilization and age groups.	27
Table S9. Odds ratios, corresponding 95% CIs, and p-values for the association between misutilization and age groups.	28
Table S10. Logistic regression results for the associations between over- and under-utilization with payor type.	29
Table S11. Odds ratios, corresponding 95% CIs, and p-values for the association between over- and under-utilization with payor type.	30
Figure S1. Network analysis for associations (using tetrachoric correlations) among all competing resources and the response resource.	31
Figure S2. Network analysis for associations (using tetrachoric correlations) of resources for patient visits with the utilization of at least one competing resource.	32
Figure S3. Decision tree analysis to isolate different patterns in resource utilization by demonstrating the value of information of competing resources with the response resource.	33

Table S1. Definitions of competing resource variables.

Competing Resources	Types
Computed Tomography Scan (CT)	CT Abdomen with & without Contrast
	CT Abdomen with Contrast
	CT Abdomen without Contrast
	CT Abdomen without Contrast Limited
	CT C-Spine with & without Contrast
	CT C-Spine with Contrast
	CT C-Spine without Contrast
	CT Cerebral Perfusion Analysis with Contrast
	CT Chest with & without Contrast
	CT Chest with Contrast
	CT Chest without Contrast
	CT Colonography Diagnostic without Contrast
	CT Facial/Sinus with & without Contrast
	CT Facial/Sinus with Contrast
	CT Facial/Sinus without Contrast
	CT Head/Brain with & without Contrast
	CT Head/Brain with Contrast
	CT Head/Brain without Contrast
	CT Heart Qn Calcium Eval without Contrast
	CT Heart Struct/Morph Congenital Eval with contrast
	CT Heart Structure/Morphology Eval with contrast
	CT L-Spine with & without Contrast
	CT L-Spine with contrast
	CT L-Spine without Contrast
	CT Lower Extremity with & without Contrast
	CT Lower Extremity with contrast
	CT Lower Extremity without Contrast
	CT Lung Cancer Screening Low Dose
	CT Neck with & without Contrast
	CT Neck with contrast
	CT Neck without Contrast
	CT Orbit/Sella/Fossa/Ear with contrast
	CT Orbit/Sella/Fossa/Ear without Contrast
	CT Pelvis & Abdomen with & without Contrast 74178
	CT Pelvis & Abdomen with contrast 74177
	CT Pelvis & Abdomen without Contrast 74176
	CT Pelvis with & without Contrast
	CT Pelvis with contrast
	CT Pelvis without Contrast
	CT T-Spine with & without Contrast
	CT T-Spine with contrast
	CT T-Spine without Contrast
	CT Unlisted Procedure

	CT Upper Extremity with contrast
	CT Upper Extremity without Contrast
	CTa Abdomen with Contrast & without If Performed
	CTa Abdominal Aorta with runoff
	CTa Chest (Noncard) With Contrast & without If Performed
	CTa Head with Contrast & without If Performed
	CTa Heart Arteries, Grafts, Structural CT/Morphological with contrast
	CTa Heart Coronaries, Morphological & Calcium Scoring
	CTa Heart Coronary Arteries & Morphology
	CTa Heart Coronary Arteries Only
	CTa Lower Extremity with Contrast & without If Performed
	CTa Neck with Contrast & without If Performed
	CTa Pelvis with Contrast & without If Performed
	CTa Upper Extremity with Contrast & without If Performed
	CTa, Abdomen & Pelvis, with Contrast, Include without Images, If Performed
Ultrasound (US)	US Abdomen
	US Abdomen Limited
	US Abdominal Aorta - Abdominal Aortic Aneurysm
	US Adrenal
	US And Duplex Doppler Kidney Transplant
	US Aorta
	US Breast Unilateral
	US Breast Unilateral Real-Time Limited
	US Breast(S)
	US Chest
	US Elastography
	US Elastography Parenchyma
	US Encephalogram
	US Extremity Nonvascular Complete
	US Extremity Nonvascular Limited
	US Gallbladder
	US Gastrointestinal Endoscopic
	US Intraoperative
	US Kidney
	US Kidney Transplant
	US Liver
	US Neck
	US Pelvis Non-Obstetric
	US Pelvis Non-Obstetric Limited
	US Retroperitoneal
	US Retroperitoneal Limited
	US Scrotum
	US Soft Tissue Head/Neck
	US Spleen

	US Target Dynamic Microbubble US Contrast Characteristic Additional Lesions
	US Target Dynamic Microbubble US Contrast Characteristic Initial Lesions
	US Thyroid
	US Transrectal Prostate
	US Transrectal Prostate Volume Study
	US Unlisted Procedure
	US Urinary Bladder
Xray (XR)	XR Abdomen 1 View Portable
	XR Chest 1 View Portable
	XR Chest 2 Views Portable
	XR Fluoroscopy 15 Min
	XR Fluoroscopy Cardiac
	XR Knee <3 Views Portable
	XR Spine 1 View Any Level Portable
	Mammogram Bilateral Diagnostic Digital
	Mammography Diagnostic Including Computer-Aided Detection Bilateral
	XR Abdomen 1 View
	XR Abdomen 2 Views
	XR Abdomen Anteroposterior Only (Kidney, Ureter, Bladder)
	XR Abdomen Anteroposterior/Decubitus/Erect
	XR Abdomen Anteroposterior/Decubitus/Erect/Chest
	XR Abdomen Anteroposterior/Oblique/Cone
	XR Abdomen Three Or More (3) Views
	XR Abscess/Fistula/Sinus Tract Study
	XR Angiogram Adrenal Unilateral
	XR Angiogram Each Additional Vessel
	XR Angiogram Extremity Bilateral
	XR Angiogram Extremity Unilateral
	XR Angiogram Iliac/Femoral Arteries Bilateral/Ipsilateral
	XR Angiogram Internal Mammary
	XR Angiogram Non-Ophthalmic Fluorescent Vascular C9733
	XR Angiogram Pelvic
	XR Angiogram Pulmonary Bilateral
	XR Angiogram Pulmonary Non-Select
	XR Angiogram Pulmonary Unilateral
	XR Angiogram Renal Bilateral
	XR Angiogram Spinal
	XR Angiogram Vertebral/Cervical Bilateral
	XR Angiogram Visceral
	XR Angioplasty Peripheral
	XR Angioplasty Venous
	XR Ankle Ap & Lateral
	XR Ankle Complete 3+ Views
	XR Aortogram Abdominal With Serialography
	XR Aortogram Bilateral Ilio/Femoral Lower Extremities Serialography

	XR Aortogram Thoracic Without Serialography
	XR Aortogram Thoracic With Serialography
	XR Bone Age Studies 77072
	XR Bone Density Dual Energy Xray Absorptiometry (DXA) Axial 77080
	XR Bone Density Dual Energy Xray Absorptiometry (DXA) Appendicular 77081
	XR Bone Survey Complete 77075
	XR Bone Survey Limited 77074
	XR C-Spine (Cervical) 4+ Views
	XR C-Spine (Cervical) Ap & Lateral
	XR C-Spine (Cervical) Complete Without Oblique/Flexion/Extension
	XR Calcaneus 2+ Views
	XR Change Tube/Drainage Catheter
	XR Chest 1 View
	XR Chest 1 View Anteroposterior
	XR Chest 1 View Posteroanterior
	XR Chest 2 Views
	XR Chest 2 Views With Apical Lordotic
	XR Chest Decubitus 1 View 71035
	XR Chest Four Or More (4) Views
	XR Chest Single View
	XR Chest Special Views
	XR Chest Three (3) Views
	XR Chest Without Obliques
	XR Cholangiogram Intraoperative
	XR Cholangiogram Postoperative (T-Tube)
	XR Cineradiography
	XR Clavicle Complete
	XR Colon Air Contrast Only 74270
	XR Colon Barium Enema
	XR Colon Barium Enema With Air Contrast
	XR Colon Hypaque Enema
	XR Colon Therapeutic Enema
	XR Cystogram 3+ Views
	XR Cystourethrogram Retrograde
	XR Cystourethrogram Voiding
	XR Dilation Biliary Duct Stricture
	XR Dilation Intraluminal Esophagus
	XR Dilation Nephron/Ureter/Urethra
	XR Elbow Anteroposterior & Lateral
	XR Elbow Complete 3+ Views
	XR Endovascular Repair Descending Thoracic Aorta Complete
	XR Endovascular Repair Infrarenal Aaa
	XR Endoscopic Retrograde Cholangiopancreatography
	XR Endoscopic Retrograde Cholangiopancreatography Biliary Only
	XR Endoscopic Retrograde Cholangiopancreatography Pancreas Only

	XR Esophagus Single-Contrast
	XR Eye Foreign Body
	XR Facial Bones Complete 3+ Views
	XR Facial Bones Limited < 3 Views
	XR Femur 1 View
	XR Femur Anteroposterior & Lateral
	XR Femur Min 2 Views
	XR Finger(S) 2+ Views
	XR Fluoroscopy < 1 Hr
	XR Fluoroscopy > 1 Hr
	XR Fluoroscopy Abscess Drainage
	XR Fluoroscopy Biopsy Intrathoracic
	XR Fluoroscopy Central Venous Access Device Place OR Rem
	XR Fluoroscopy Needle Place Biopsy/Aspiration/Injection
	XR Fluoroscopy Needle/Catheter Tip Localization
	XR Fluoroscopy Procedure Unlisted
	XR Foot Anteroposterior & Lateral
	XR Foot Complete 3+ Views
	XR Forearm Anteroposterior & Lateral
	XR Hand Complete 3+ Views
	XR Hand Limited 2 Views
	XR Hip Bilateral With Pelvis 2 Views
	XR Hip Bilateral With Pelvis 3-4 Views
	XR Hip Bilateral With Pelvis Min 5 Views
	XR Hip Intraoperative
	XR Hip Unilateral Complete 2+ Views
	XR Hip Unilateral Limited 1 View
	XR Hip Unilateral With Pelvis 1 View
	XR Hip Unilateral With Pelvis 2-3 Views
	XR Hip Unilateral With Pelvis Min 4 Views
	XR Hips Bilateral With Pelvis 4+ Views
	XR Humerus 2+ Views
	XR Introduction of Long Gastrointestinal Tube
	XR Introduction of Renal Pelvis Catheter
	XR Introduction of Ureteral Catheter/Stent
	XR Inferior Vena Cava Filter Placement
	XR Joint Survey 1 View 77077
	XR Knee <3 Views
	XR Knee 3 Views
	XR Knee Anteroposterior Standing Bilateral
	XR Knee Complete 4+ Views
	XR L-Spine (Lumbar) Complete With Bending Views
	XR L-Spine (Lumbar) Complete Without obliques
	XR L-Spine (Lumbar) 3 Views
	XR L-Spine (Lumbar) Anteroposterior & Lateral
	XR L-Spine (Lumbar) Bending Views Only

	XR Lymphangiogram Pelvic/Abdomen Bilateral
	XR Lymphangiogram Pelvic/Abdomen Unilateral
	XR Mammary Ductogram Single 77053
	XR Mandible < 4 Views
	XR Mandible Complete 4+ Views
	XR Myelogram L-Spine (Lumbar)
	XR Myelogram T-Spine (Thoracic)
	XR Myelogram Two Or More Regions
	XR Nasal Bones Complete 3+ Views
	XR Navicular Study
	XR Neck Soft Tissue
	XR Orbits Complete 4+ Views
	XR Orthopantomogram
	XR Pelvis 1 Or 2 Views
	XR Pelvis Complete 3+ Views
	XR Percutaneous Vertebroplasty/Vertebral Augmentation/Sacroplasty 72291
	XR Pharynx/Esophagus Swallow Cine/Video
	XR Placement Biliary Drainage Catheter
	XR Placement Gastrostomy Tube
	XR Portogram With Hemodynamics
	XR Portogram Without Hemodynamics
	XR Renal Cyst Study
	XR Ribs Bilateral 3+ Views
	XR Ribs Bilateral With Chest 4+ Views
	XR Ribs Unilateral 2 Views
	XR Ribs Unilateral With Chest 3+ Views
	XR Sacrum/Coccyx 2+ Views
	XR Scapula Complete
	XR Setup
	XR Shoulder Complete 2+ Views
	XR Shoulder Limited 1 View
	XR Shuntogram Nonvascular
	XR Sacroiliac Joints < 3 Views
	XR Sacroiliac Joints 3+ Views
	XR Sinuses Complete 3+ Views
	XR Sinuses Limited < 3 Views
	XR Skull 3 Views
	XR Skull Complete 4+ Views
	XR Skull Limited < 4 Views
	XR Small Intestine Bowel
	XR Speech Evaluation Complex
	XR Spine 1 View Any Level
	XR Spine Entire Lumbar Thoracic Skull Cervical 2 -3 Views
	XR Spine Entire Lumbar Thoracic Skull Cervical One View
	XR Sternum 2+ Views

	XR Stress View(S) 77071
	XR Surgical Specimen
	XR T-L-Spine (Thoracolumbar) Anteroposterior & Lateral
	XR T-Spine (Thoracic) Anteroposterior & Lateral
	XR T-Spine (Thoracic) Complete
	XR T-Spine (Thoracic) With Swimmers View
	XR Teeth Complete
	XR Temporomandibular Joint Bilateral
	XR Tibia/Fibula Anteroposterior & Lateral
	XR Toe(S) 2+ Views
	XR Transcathether Angiogram Existing Catheter
	XR Transcatheter Retrieval Foreign Body
	XR Transcatheter Therapy Embolization
	XR Transcatheter Therapy Infusion
	XR Upper gastrointestinal With Air Contrast With Kidney, Ureter, Bladder
	XR Upper gastrointestinal With Air Contrast Without Kidney, Ureter, Bladder
	XR Upper gastrointestinal With Air Contrast With Small Intestine Bowel
	XR Upper gastrointestinal With Kidney, Ureter, Bladder
	XR Upper gastrointestinal Without Kidney, Ureter, Bladder
	XR Upper gastrointestinal With Small Intestine Bowel
	XR Unlisted Procedure
	XR Urogram Antegrade
	XR Urogram Intravenous pyelogram (IVP)
	XR Urogram Retrograde
	XR Urogram With Nephrotomogram
	XR Venocavagram Inferior
	XR Venocavagram Inferior Each Additional
	XR Venocavagram Superior
	XR Venocavagram Superior Each Additional
	XR Venogram Extremity Bilateral
	XR Venogram Extremity Unilateral
	XR Wrist Anteroposterior & Lateral
	XR Wrist Complete 3+ Views
Special Imaging	CT Followup/Limited
	CT Guided Abscess Drainage
	CT Guided Cyst Aspiration
	CT Guided Localization Stereotactic 77011
	CT Guided Needle Placement 77012
	CT Guided Radiation Therapy Placement 77014
	CT Guided Tissue Ablation
	CT Reconstruction
	CT Reconstruction With Contrast
	CT Reconstruction Without Contrast
	MRI Reconstruction
	NM Abscess Localize Limited

	NM Abscess Localize Single-photon emission computed tomography
	NM Abscess Localize Whole Body
	NM Computer Analysis < 30 Min
	NM Injection Radiopharmaceutical Localization Non-Imaging Study, Intravenous
	US Guide & Monitor of Parenchymal Tissue Ablation
	US Guide for Vascular Access
	US Guide Interstitial Radioelement Application
	US Guide Intraoperative
	US Guided Abscess Drainage
	US Guided Compression Repair
	US Guided Cyst Aspiration
	US Guided Needle Biopsy
	US Guided Pericardiocentesis
	US Guided Thoracentesis/Paracentesis
	XR, CT, MRI, US 3D Render Any Tomosynthesis Modal Without Postprocess
	XR, CT, MRI, US 3D Render Any Tomosynthesis Modal With Postprocess
Nuclear Medicine (NM)	NM Bone 3 Phase
	NM Bone Limited
	NM Bone Marrow Limited
	NM Bone Marrow Multiple
	NM Bone Multiple
	NM Bone Single-photon emission computed tomography
	NM Bone Whole Body
	NM Bowel
	NM Brain < 4 Static Views With Flow
	NM Brain 4 Static Views With Flow
	NM Cardiac Blood Pool Multiple
	NM Cardiac Blood Pool Single
	NM Cisternogram
	NM Cardiovascular Procedure Unlisted
	NM Diagnostic Procedure Unlisted 78999
	NM Gastric Emptying
	NM Gastrointestinal Blood Loss
	NM Genitourinary Procedure Unlisted
	NM Hepatobiliary Ducts
	NM Kidney Flow & Function Sing With Pharmacy 78708
	NM Kidney Scan Morphology
	NM Kidney Scan With Flow
	NM Kidney Scan With Flow & Function
	NM Kidney With Flow & Function Multi With & Without Pharmaceuticals
	NM Kidney With Flow & Function Single With Pharmaceuticals
	NM Liver & Spleen
	NM Localization Tumor Planar Single Area Single Day Imaging
	NM Localization Tumor Planar Whole Body Single Day Imaging

	NM Localization Tumor Single-photon emission computed tomography Single Area Single Day Imaging
	NM Lymph System
	NM Musculoskeletal Procedure Unlisted
	NM Myocardial Infarction
	NM Myocardial Infarction Single-photon emission computed tomography
	NM Myocardial Infarction With Ejection Fraction 1st Pass
	NM Myocardial Perfusion Multiple
	NM Myocardial Perfusion Multiple Planar With Wall,Ejection Fraction, Rest/Stress
	NM Myocardial Perfusion Multiple Single-photon emission computed tomography
	NM Myocardial Perfusion Single Single-photon emission computed tomography With Wall,Ejection Fraction, Rest/Stress
	NM Myocardial Perfusion Single Single-photon emission computed tomography
	NM Myocardial Perfusion Wall Motion
	NM Parathyroid
	NM Pulmonary Perfusion Part
	NM Pulmonary Perfusion Part With Ventilation
	NM Pulmonary Perfusion Part With Ventilation R&W
	NM Pulmonary Perfusion Particulate With Aerosol Ventilation
	NM Pulmonary Vent Gas Single R&W
	NM Pulmonary Vent/Perfusion Difference
	NM Respiratory Procedure Unlisted
	NM Shunt Evaluation
	NM Thyroid Scan With Uptake Single
	NM Urea Breath Test C-14 Analysis
	NM Vascular Flow
	NM Whole Blood Volume
Magnetic Resonance Imaging & Magnetic Resonance Angiography (MRI & MRA)	MRA Abdomen
	MRA Abdomen With & Without Contrast
	MRA Chest
	MRA Head With & Without Contrast
	MRA Head With contrast
	MRA Head Without Contrast
	MRA Head/Neck
	MRA Lower Extremity
	MRA Neck With & Without Contrast
	MRA Neck With contrast
	MRA Neck Without Contrast
	MRA Pelvis
	MRA Spine
	MRI Abdomen With & Without Contrast
	MRI Abdomen With contrast
	MRI Abdomen Without Contrast

	MRI Brain During Intracranial Procedure With & Without Contrast
	MRI Brain Function No MD/Psychologist Administration
	MRI Brain With & Without Contrast
	MRI Brain With contrast
	MRI Brain Without Contrast
	MRI C-Spine With & Without Contrast
	MRI C-Spine With contrast
	MRI C-Spine Without Contrast
	MRI Cardiac Function Complete with Flow and Velocity Quantification & Stress Imaging
	MRI Cardiac Function Complete
	MRI Cardiac Function Complete With Stress Imaging
	MRI Cardiac Velocity Flow Mapping
	MRI Cardiac Without Contrast
	MRI Chest With & Without Contrast
	MRI Chest Without Contrast
	MRI L-Spine With & Without Contrast
	MRI L-Spine With contrast
	MRI L-Spine Without Contrast
	MRI L-Spine Without Contrast Limited
	MRI Lower Extremity Joint With & Without Contrast
	MRI Lower Extremity Joint With contrast
	MRI Lower Extremity Joint Without Contrast
	MRI Lower Extremity With & Without Contrast
	MRI Lower Extremity With contrast
	MRI Lower Extremity Without Contrast
	MRI Orbit/Face/Neck With & Without Contrast
	MRI Orbit/Face/Neck With contrast
	MRI Orbit/Face/Neck Without Contrast
	MRI Pelvis With & Without Contrast
	MRI Pelvis With contrast
	MRI Pelvis Without Contrast
	MRI Spectroscopy
	MRI T-Spine With & Without Contrast
	MRI T-Spine With contrast
	MRI T-Spine Without Contrast
	MRI Unlisted Procedure
	MRI Upper Extremity Joint With & Without Contrast
	MRI Upper Extremity Joint Without Contrast
	MRI Upper Extremity With & Without Contrast
	MRI Upper Extremity With contrast
	MRI Upper Extremity Without Contrast
Miscellaneous Imaging	CT Extended Room Time 30 Minutes
	Injection Sinus US Tract/Sinogram
	US Callback Fee
	US Follow Up

	US Portable Fee
	XR Arteriogram Setup
	XR Consultation
	XR Film Copy
	XR Outside Facility
	XR Portable Fee
	XR Room Time 1st Hour
	XR Room Time 30 Minutes
	XR Room Time Additional 30 Minutes
	XR Statim Fee
	XR Technician in OR 30 Minutes
	XR Technician in OR Flat Rate

Table S2. Multivariate logistic regression analysis results with coefficients (Coef), standard errors (SE), and p-values (p) for the three models: (a) patient characteristics and competing resource utilization; (b) patient characteristics only; and (c) competing resource utilization only.

	**Model 1: Patient and competing resource characteristics**			**Model 2: Patient characteristics only**			**Model 3: Competing resource characteristics only**		
	**Coef**	**SE**	**p**	**Coef**	**SE**	**p**	**Coef**	**SE**	**p**
(Intercept)	-2.4499	0.6054	<0.0001	-1.7827	0.5838	0.0023	-2.9967	0.0305	<0.0001
Length of Stay	0.0416	0.0027	<0.0001	0.0555	0.0027	<0.0001			
History of Malignancy	0.0383	0.0582	0.5108	0.0474	0.0573	0.4087			
Procedural Cost Type	0.1248	0.0320	<0.0001	0.1889	0.0314	<0.0001			
Secondary ICD Classification	-0.7067	0.0907	<0.0001	-0.8674	0.0854	<0.0001			
**Age (Reference: ≤45)**									
46-50	-0.4971	0.3848	0.1964	-0.5748	0.3731	0.1234			
51-55	-0.3240	0.3352	0.3338	-0.4914	0.3227	0.1278			
56-60	-0.2266	0.3224	0.4821	-0.3333	0.3094	0.2814			
61-65	-0.2135	0.3194	0.5039	-0.3519	0.3064	0.2508			
66-70	-0.1912	0.3201	0.5503	-0.3231	0.3071	0.2927			
71-75	-0.2948	0.3204	0.3574	-0.4180	0.3074	0.1739			
76-80	-0.2710	0.3207	0.3981	-0.4090	0.3077	0.1838			
81-85	-0.2844	0.3213	0.3760	-0.4094	0.3083	0.1843			
> 85	-0.1945	0.3215	0.5453	-0.3378	0.3085	0.2735			
**Race (Reference: White)**									
American Indian	0.1365	0.2327	0.5575	0.1112	0.2266	0.6236			
Asian	-0.1835	0.1208	0.1285	-0.1774	0.1185	0.1344			
Black	-0.0258	0.0407	0.5264	-0.0061	0.0402	0.8786			
Other	-0.1762	0.0679	0.0095	-0.1834	0.0668	0.0061			
Pacific Islander	-0.0257	0.2380	0.9141	-0.0287	0.2366	0.9034			
Unknown	-0.0786	0.1161	0.4986	-0.0895	0.1155	0.4383			
**Payor Type (Reference: Medicare Traditional)**									
Charity or Indigent	0.3617	0.3674	0.3248	0.3196	0.3575	0.3713			
Commercial Indemnity	-0.1773	0.0971	0.0679	-0.1877	0.0956	0.0496			
Direct Employer Contract	-0.0862	0.4826	0.8582	-0.0936	0.4792	0.8451			

Managed Care Capitated	-0.4570	0.3624	0.2073	-0.4792	0.3617	0.1852			
Managed Care Non Capitated	-0.2738	0.0671	<0.0001	-0.2951	0.0662	<0.0001			
Medicaid Managed Care Capitated	0.0293	0.2130	0.8904	-0.0105	0.2089	0.9599			
Medicaid Managed Care Non Capitated	-0.3138	0.1196	0.0087	-0.3404	0.1177	0.0038			
Medicaid Traditional	-0.0322	0.1226	0.7926	-0.0173	0.1209	0.8863			
Medicare Managed Care Capitated	-0.1977	0.0633	0.0018	-0.2909	0.0624	<0.0001			
Medicare Managed Care Non Capitated	-0.0103	0.0386	0.7895	0.0052	0.0381	0.8919			
Other	-0.5047	0.1623	0.0019	-0.7581	0.1615	<0.0001			
Other Government Payers	-0.0402	0.1031	0.6969	-0.0804	0.1019	0.4304			
Self Pay	0.1834	0.1560	0.2397	0.2020	0.1521	0.1842			
Workers Compensation	-0.2284	0.5362	0.6701	-0.1548	0.5322	0.7711			
**Point of Origin (Reference: Non-Healthcare Facility (Physician Referral))**									
Clinic	-0.1323	0.0538	0.0139	-0.1488	0.0530	0.0050			
Court/Law Enforcement	0.0823	0.5770	0.8866	0.0503	0.5766	0.9304			
Information Not Available	-0.4294	0.1975	0.0297	-0.6701	0.1957	0.0006			
Transfer from a Hospital (Different Facility)	-0.6268	0.0748	<0.0001	-0.6538	0.0735	<0.0001			
Transfer from an SNF or ICF	-0.0153	0.1091	0.8883	-0.0595	0.1074	0.5799			
Transfer from Ambulatory Surgical Center	-0.9666	0.7443	0.1940	-1.0202	0.7445	0.1706			
Transfer from Another Healthcare Facility	-0.3345	0.1669	0.0450	-0.3357	0.1631	0.0396			
Transfer from Hospice and is Under a Hospice Plan of Care or Enrolled in a Hospice Program	-1.3402	1.1876	0.2591	-1.3238	1.1502	0.2498			
Transfer from Hospital Inpatient in the Same Facility Resulting in a Separate Claim to the Payor	-0.0738	0.2173	0.7343	-0.1320	0.2141	0.5375			
**Discharge Status (Reference: Court/Law Enforcement)**									
Expired	0.6210	0.5174	0.2300	0.4947	0.4997	0.3222			
Home Health Organization	0.3047	0.5142	0.5534	0.2308	0.4965	0.6420			
Home or Self Care	0.2941	0.5133	0.5667	0.1847	0.4956	0.7094			
Hospice Home	0.5320	0.5181	0.3045	0.3694	0.5003	0.4604			
Hospice Medical Facility	0.6450	0.5196	0.2144	0.5011	0.5019	0.3181			
Left Against Medical Advice	0.3929	0.5537	0.4780	0.2319	0.5369	0.6658			
Other	0.5767	0.5324	0.2788	0.3965	0.5153	0.4416			
Transferred to a Long Term Care Hospital	0.5096	0.5402	0.3455	0.4014	0.5226	0.4424			
Transferred to Another Rehabilitation Facility	0.0639	0.5221	0.9025	-0.0570	0.5044	0.9101			

Transferred to ICF	0.1109	0.5598	0.8429	0.0325	0.5420	0.9522			
Transferred to Other Facility	0.6383	0.5241	0.2233	0.5416	0.5065	0.2849			
Transferred to SNF	0.3388	0.5146	0.5103	0.2388	0.4969	0.6308			
Transferred to Swing Bed	0.3723	0.5799	0.5209	0.3162	0.5620	0.5737			
**Medicare Severity Diagnosis Related Groups (MS-DRGs) (Reference: Other)**									
Acute Myocardial Infarction (Discharged Alive)	-0.9293	0.1883	<0.0001	-0.8425	0.1872	<0.0001			
Cardiac Arrhythmia Conduction Disorders	-1.0121	0.1980	<0.0001	-0.9811	0.1972	<0.0001			
Esophagitis Gastroenteritis Miscellaneous Digest Disorders	0.8304	0.1103	<0.0001	0.7292	0.1090	<0.0001			
Gastrointestinal Hemorrhage	0.1241	0.1046	0.2354	0.0548	0.1030	0.5947			
Heart Failure (Shock)	-0.7887	0.1168	<0.0001	-0.7221	0.1156	<0.0001			
Infectious Parasitic Diseases W.O.R. Procedure	0.8572	0.0997	<0.0001	0.9308	0.0978	<0.0001			
Intracranial Hemorrhage or Cerebral Infarction	-0.5247	0.1812	0.0038	-0.7486	0.1788	<0.0001			
Kidney/ Urinary Tract Infections	0.8723	0.0872	<0.0001	0.8424	0.0859	<0.0001			
Major Joint Replacement or Reattachment of lower extremity	-1.4404	0.2318	<0.0001	-1.3702	0.2305	<0.0001			
Major Male Pelvic Procedures	-1.8899	0.1163	<0.0001	-2.2828	0.1113	<0.0001			
Malignancy of Male Reproductive System	0.0520	0.1137	0.6473	-0.0141	0.1093	0.8972			
Miscellaneous Disorders of Nutrition Metabolism Fluids Electrolytes	-0.4937	0.1340	0.0002	-0.5570	0.1328	<0.0001			
Other Kidney/Urinary Tract Diagnoses	0.7527	0.0679	<0.0001	0.7444	0.0665	0.0000			
Pathological Fractures Musculoskeletal Connective Tissue Malignancy	-0.3411	0.1261	0.0068	-0.3859	0.1240	0.0019			
Per-cutaneous Cardiovascular procedure with Stent	-1.2149	0.2403	<0.0001	-1.0885	0.2377	<0.0001			
Renal Failure	0.9536	0.0641	<0.0001	1.0765	0.0619	<0.0001			
Septicemia or Severe Sepsis W.O MV 96 Hours	0.5185	0.0488	<0.0001	0.5454	0.0480	<0.0001			
Simple Pneumonia Pleurisy	-0.6407	0.1358	<0.0001	-0.6383	0.1346	<0.0001			
**Comorbidities**									
Diabetes - Complicated	-0.0434	0.0394	0.2715	-0.0369	0.0389	0.3431			
Diabetes - Uncomplicated	0.1198	0.0501	0.0167	0.1098	0.0495	0.0265			
Other Neurological Disorders	-0.1569	0.0441	0.0004	-0.1880	0.0430	<0.0001			
Congestive Heart Failure	-0.2358	0.0468	<0.0001	-0.1907	0.0460	<0.0001			
Obesity	0.0203	0.0485	0.6761	0.0164	0.0479	0.7319			
Fluid and Electrolyte Disorders	0.4147	0.0337	<0.0001	0.4832	0.0332	<0.0001			
Lymphoma	-0.0650	0.1174	0.5803	-0.0424	0.1155	0.7135			
Chronic Pulmonary Disease	-0.0990	0.0383	0.0098	-0.0991	0.0378	0.0088			
Paralysis	-0.5102	0.1212	<0.0001	-0.6019	0.1201	<0.0001			

Cardiac Arrhythmia	-0.1024	0.0354	0.0039	-0.0841	0.0349	0.0160			
Renal Failure	0.7217	0.0477	<0.0001	0.7914	0.0470	<0.0001			
Hypertension - Complicated	0.1432	0.0504	0.0045	0.1476	0.0497	0.0030			
Hypertension - Uncomplicated	0.0628	0.0425	0.1400	0.0723	0.0420	0.0855			
Pulmonary Circulation Disorders	-0.2162	0.0678	0.0014	-0.2032	0.0670	0.0024			
Coagulopathy	-0.0356	0.0472	0.4496	-0.0176	0.0464	0.7043			
Weight Loss	-0.0474	0.0434	0.2752	-0.0378	0.0429	0.3777			
Liver Disease	0.2866	0.0644	<0.0001	0.4023	0.0630	<0.0001			
Metastatic Cancer	0.1851	0.0335	<0.0001	0.2332	0.0326	<0.0001			
Peripheral Vascular Disorders	-0.0526	0.0498	0.2908	-0.0471	0.0491	0.3372			
Depression	-0.0872	0.0517	0.0913	-0.1017	0.0511	0.0463			
Rheumatoid Arthritis Collagen	-0.1183	0.1136	0.2976	-0.1042	0.1118	0.3517			
Hypothyroidism	-0.0335	0.0510	0.5110	-0.0422	0.0503	0.4016			
Anemia Deficiency	0.1027	0.0589	0.0810	0.1236	0.0580	0.0331			
Valvular Disease	-0.1658	0.0558	0.0030	-0.1472	0.0551	0.0076			
Drug Abuse	-0.1257	0.1039	0.2265	-0.1223	0.1025	0.2326			
Alcohol Abuse	-0.1300	0.0823	0.1141	-0.0918	0.0811	0.2577			
**Competing Resources**									
CT Scans (Excluding Outcome Resource)	-0.1680	0.0340	<0.0001				0.0557	0.0315	0.0773
Miscellaneous	0.1196	0.0943	0.2048				0.1839	0.0898	0.0405
MRI and MRA	-0.1815	0.0503	0.0003				-0.1860	0.0460	<0.0001
Nuclear Medicine	0.7237	0.0514	<0.0001				0.8431	0.0473	<0.0001
Special Imaging Techniques - All Imaging	0.4301	0.0484	<0.0001				0.6484	0.0449	<0.0001
Ultrasound	0.5764	0.0366	<0.0001				1.1377	0.0332	<0.0001
X - Ray	0.4844	0.0403	<0.0001				0.9148	0.0363	<0.0001

Table S3. Binary representation of competing resources indicating the usage of different combinations of resources (0=not used; 1=used) along with the count of cases where the outcome resource is used and not used.

Binary Representation of Covariates[1]	Cases with Observed Combination, N (%)	Cases where Response Resource was Not Used, N (%)	Cases where Response Resource was Used, N (%)
0000000	17,635 (34.5)	17,052 (96.69)	583 (3.31)
0000001	14 (0.03)	11 (78.57)	3 (21.43)
0000010	147 (0.29)	92 (62.59)	55 (37.41)
0000100	625 (1.22)	483 (77.28)	142 (22.72)
0000101	5 (0.01)	1 (20)	4 (80)
0000110	37 (0.07)	15 (40.54)	22 (59.46)
0001000	265 (0.52)	244 (92.08)	21 (7.92)
0001001	1 (0)	1 (100)	0 (0)
0001010	12 (0.02)	10 (83.33)	2 (16.67)
0001100	43 (0.08)	32 (74.42)	11 (25.58)
0001101	1 (0)	1 (100)	0 (0)
0001110	5 (0.01)	2 (40)	3 (60)
0010000	178 (0.35)	146 (82.02)	32 (17.98)
0010001	5 (0.01)	1 (20)	4 (80)
0010010	5 (0.01)	3 (60)	2 (40)
0010100	32 (0.06)	20 (62.5)	12 (37.5)
0010101	3 (0.01)	2 (66.67)	1 (33.33)
0010110	4 (0.01)	1 (25)	3 (75)
0010111	1 (0)	1 (100)	0 (0)
0011000	19 (0.04)	17 (89.47)	2 (10.53)
0011010	4 (0.01)	3 (75)	1 (25)
0011100	5 (0.01)	1 (20)	4 (80)
0011110	2 (0)	0 (0)	2 (100)
0100000	9,485 (18.56)	8,336 (87.89)	1,149 (12.11)
0100001	165 (0.32)	149 (90.3)	16 (9.7)
0100010	393 (0.77)	313 (79.64)	80 (20.36)
0100011	11 (0.02)	9 (81.82)	2 (18.18)
0100100	1,571 (3.07)	1,140 (72.57)	431 (27.43)
0100101	38 (0.07)	23 (60.53)	15 (39.47)
0100110	144 (0.28)	71 (49.31)	73 (50.69)
0100111	9 (0.02)	3 (33.33)	6 (66.67)
0101000	450 (0.88)	411 (91.33)	39 (8.67)
0101001	10 (0.02)	9 (90)	1 (10)
0101010	45 (0.09)	35 (77.78)	10 (22.22)
0101100	127 (0.25)	95 (74.8)	32 (25.2)
0101101	4 (0.01)	3 (75)	1 (25)
0101110	19 (0.04)	11 (57.89)	8 (42.11)

0110000	556 (1.09)	441 (79.32)	115 (20.68)
0110001	21 (0.04)	15 (71.43)	6 (28.57)
0110010	52 (0.1)	30 (57.69)	22 (42.31)
0110011	5 (0.01)	0 (0)	5 (100)
0110100	189 (0.37)	108 (57.14)	81 (42.86)
0110101	12 (0.02)	8 (66.67)	4 (33.33)
0110110	25 (0.05)	12 (48)	13 (52)
0110111	3 (0.01)	1 (33.33)	2 (66.67)
0111000	52 (0.1)	46 (88.46)	6 (11.54)
0111001	2 (0)	2 (100)	0 (0)
0111010	12 (0.02)	7 (58.33)	5 (41.67)
0111011	1 (0)	1 (100)	0 (0)
0111100	27 (0.05)	18 (66.67)	9 (33.33)
0111101	1 (0)	0 (0)	1 (100)
0111110	4 (0.01)	2 (50)	2 (50)
1000000	1,725 (3.38)	1,595 (92.46)	130 (7.54)
1000001	5 (0.01)	4 (80)	1 (20)
1000010	165 (0.32)	133 (80.61)	32 (19.39)
1000011	1 (0)	1 (100)	0 (0)
1000100	241 (0.47)	205 (85.06)	36 (14.94)
1000101	3 (0.01)	1 (33.33)	2 (66.67)
1000110	26 (0.05)	15 (57.69)	11 (42.31)
1000111	1 (0)	0 (0)	1 (100)
1001000	504 (0.99)	483 (95.83)	21 (4.17)
1001001	3 (0.01)	3 (100)	0 (0)
1001010	59 (0.12)	51 (86.44)	8 (13.56)
1001100	58 (0.11)	46 (79.31)	12 (20.69)
1001110	12 (0.02)	8 (66.67)	4 (33.33)
1010000	100 (0.2)	81 (81)	19 (19)
1010001	6 (0.01)	5 (83.33)	1 (16.67)
1010010	39 (0.08)	28 (71.79)	11 (28.21)
1010011	2 (0)	2 (100)	0 (0)
1010100	21 (0.04)	16 (76.19)	5 (23.81)
1010110	14 (0.03)	9 (64.29)	5 (35.71)
1010111	1 (0)	1 (100)	0 (0)
1011000	64 (0.13)	60 (93.75)	4 (6.25)
1011001	2 (0)	1 (50)	1 (50)
1011010	24 (0.05)	16 (66.67)	8 (33.33)
1011011	2 (0)	2 (100)	0 (0)
1011100	12 (0.02)	10 (83.33)	2 (16.67)
1011110	4 (0.01)	2 (50)	2 (50)
1100000	8,063 (15.78)	7,065 (87.62)	998 (12.38)
1100001	211 (0.41)	176 (83.41)	35 (16.59)
1100010	577 (1.13)	472 (81.8)	105 (18.2)
1100011	17 (0.03)	13 (76.47)	4 (23.53)
1100100	1,779 (3.48)	1,306 (73.41)	473 (26.59)

1100101	65 (0.13)	48 (73.85)	17 (26.15)
1100110	196 (0.38)	102 (52.04)	94 (47.96)
1100111	6 (0.01)	4 (66.67)	2 (33.33)
1101000	1,920 (3.76)	1,738 (90.52)	182 (9.48)
1101001	51 (0.1)	44 (86.27)	7 (13.73)
1101010	238 (0.47)	190 (79.83)	48 (20.17)
1101011	5 (0.01)	3 (60)	2 (40)
1101100	421 (0.82)	311 (73.87)	110 (26.13)
1101101	17 (0.03)	14 (82.35)	3 (17.65)
1101110	93 (0.18)	60 (64.52)	33 (35.48)
1101111	4 (0.01)	4 (100)	0 (0)
1110000	708 (1.39)	562 (79.38)	146 (20.62)
1111001	21 (0.04)	18 (85.71)	3 (14.29)
1111010	91 (0.18)	70 (76.92)	21 (23.08)
1111011	11 (0.02)	7 (63.64)	4 (36.36)
1111100	108 (0.21)	68 (62.96)	40 (37.04)
1111101	5 (0.01)	3 (60)	2 (40)
1111110	51 (0.1)	27 (52.94)	24 (47.06)
1111111	4 (0.01)	3 (75)	1 (25)

[1]Binary seven-digit value elaboration (left to right): 1st digit = Other CT scans excluding outcome resource; 2nd digit = X-rays; 3rd digit = Special imaging techniques; 4th digit = MRI & MRA; 5th digit = Ultrasound; 6th digit = Nuclear Medicine; 7th digit = Miscellaneous

Table S4. Patient characteristics for the top and bottom five patient-visits where predicted resource usage probabilities and actual resource utilization are not aligned.

Patient ID	345	37368	12962	25049	10468	5412	35121	16911	11910	9292
**Use of CT of Pelvis and Abdomen Without Contrast (Response Resource)**										
Actual	Yes	Yes	Yes	Yes	Yes	No	No	No	No	No
Predicted	No	No	No	No	No	Yes	Yes	Yes	Yes	Yes
Predicted Probability	0.0036	0.0073	0.008	0.0087	0.0094	0.9627	0.9688	0.978	0.9824	0.9933
**Patient Characteristics**										
Length of Stay	2	3	1	3	1	100	103	134	166	135
Standard Payor Description	Managed Care Non-Cap	Other	Medicaid Managed Care Non-Cap	Commercial Indemnity	Managed Care Non-Cap	Self-Pay	Medicare Managed Care Non-Cap	Other Government Payers	Medicare Managed Care Non-Cap	Commercial Indemnity
ICD Class for Prostate Cancer Diagnosis	Principal	Principal	Principal	Principal	Principal	Secondary	Secondary	Secondary	Secondary	Secondary
History of Malignancy	No	No	No	No	No	No	No	Yes	No	Yes
Age Group	46-50	66-70	56-60	56-60	66-70	61-65	81-85	71-75	71-75	51-55
Race	White	White	White	White	White	White	Black	White	White	Black
Point of Origin	Transfer from Ambulatory Surgery Center	Clinic	Clinic	Clinic	Clinic	Non-Healthcare Facility	Non-Healthcare Facility	Non-Healthcare Facility	Non-Healthcare Facility	Non-Healthcare Facility
Primary Reason for Hospital Visit (MS-DRG [1])	Major Male Pelvic Procedures	Major Male Pelvic Procedures	Major Male Pelvic Procedures	Major Male Pelvic Procedures	Major Male Pelvic Procedures	Other Kidney Urinary Tract	Other	Cardiac Arrhythmia Conductio	Other	Other

						Diagnosi s		n Disorders		
Discharge Status	Home/Self Care	Home/Sel f Care	Home/Sel f Care	Home/Self Care	Home/Sel f Care	Other	Expired	Transferre d to SNF	Transferre d to SNF	Expired
Cost Type	Procedura l	Cost to Charge Ratio	Cost to Charge Ratio	Cost to Charge Ratio	Procedur al	Cost to Charge Ratio	Procedur al	Procedural	Procedura l	Procedura l
Length of Stay	2	3	1	3	1	100	103	134	166	135
Standard Payor Description	Managed Care Non-Cap	Other	Medicaid Managed Care Non-Cap	Commerci al Indemnity	Managed Care Non-Cap	Self-Pay	Medicare Managed Care Non-Cap	Other Governme nt Payers	Medicare Managed Care Non-Cap	Commerci al Indemnity
ICD Class	Principal	Principal	Principal	Principal	Principal	Secondar y	Secondar y	Secondary	Secondary	Secondary
**Comorbidities**										
Diabetes Uncomplicate d	No	Yes	No	No	No	No	No	No	No	No
Other Neurological Disorders	No	No	No	No	No	No	Yes	Yes	Yes	No
Congestive Heart Failure	No	No	No	No	No	No	Yes	Yes	No	No
Obesity	No	No	No	Yes	No	No	No	No	No	No
Fluid and Electrolyte Disorders	No	No	No	No	No	No	Yes	Yes	No	Yes
Lymphoma	No	No	No	No	No	No	No	No	No	No
Chronic Pulmonary Disease	No	No	No	Yes	Yes	No	Yes	No	No	No
Paralysis	No	No	No	No	No	No	No	No	No	No

Cardiac Arrhythmia	No	No	Yes	No	No	Yes	Yes	Yes	No	No
Renal Failure	No	No	No	No	No	Yes	Yes	Yes	No	Yes
Hypertension Uncomplicated	No	Yes	No	No	No	No	No	No	No	No
Pulmonary Circulation Disorders	No	No	No	No	No	No	No	No	No	No
Coagulopathy	No	No	Yes	No	No	No	No	No	No	No
Weight Loss	No	No	No	No	No	No	Yes	No	Yes	Yes
Liver Disease	No	No	No	No	No	No	Yes	No	No	No
Metastatic Cancer	No	No	No	No	No	Yes	Yes	No	No	No
Diabetes Complicated	No	No	No	No	No	No	Yes	No	No	Yes
Peripheral Vascular Disorders	Yes	No	No	No	No	No	No	No	No	No
Depression	No	No	No	No	Yes	No	No	No	No	Yes
Hypertension Complicated	No	No	No	No	No	Yes	Yes	Yes	No	No
Rheumatoid Arthritis Collagen	No	No	No	No	No	No	No	No	No	No
Hypothyroidism	No	No	No	Yes	No	No	No	No	No	No
Deficiency Anemia	No	No	No	No	No	No	Yes	No	No	No
Valvular Disease	No	No	No	No	No	No	No	No	No	No
Drug Abuse	No	No	No	No	No	No	No	No	No	No
Alcohol Abuse	No	No	No	No	No	No	No	No	No	No

**Imaging Modalities Used With Response Resource**										
CT	No	Yes	No	Yes	No	No	Yes	Yes	Yes	Yes
X-ray	No	No	No	No	No	No	Yes	Yes	Yes	Yes
Special Imaging Techniques	No	No	No	No	No	No	No	No	No	Yes
MRI/MRA	No	Yes	No	No	No	No	Yes	Yes	No	No
Ultrasound	No	No	No	No	No	No	Yes	Yes	No	Yes
Nuclear Medicine	No	No	No	No	No	No	No	Yes	No	No
Miscellaneous	No	No	No	No	No	No	No	No	No	No

[1] MS-DRG: Medical Severity-Diagnosis Related Grouping

Table S5. Examples of the top and bottom 5 facilities, among those with at least 30 patients, based on the percentage of mis- (left), over- (middle), and under- (right) utilization of resources along with the count of patients in each facility receiving the outcome imaging resource.

Facility ID	Percentage Utilization	Patient Count	Facility ID	Percentage Utilization	Patient Count	Facility ID	Percentage Utilization	Patient Count
**Lowest Mis-utilization**			**Lowest Over-utilization**			**Lowest Under-utilization**		
321	0	32	37	0	32	321	0	32
456	0	49	45	0	35	456	0	49
466	0	55	87	0	42	466	0	55
1022	0	45	93	0	54	949	0	50
1063	0	36	111	0	36	1022	0	45
**Highest Mis-utilization**			**Highest Over-utilization**			**Highest Under-utilization**		
783	41.86	43	706	20.51	39	329	32.26	31
718	42.86	49	628	22.50	40	962	33.87	62
1183	45.00	40	1106	23.33	30	650	37.23	94
962	45.16	62	680	26.47	34	782	38.46	39
1221	46.67	30	1221	30	30	718	42.86	49

Table S6. Logistic regression results for the association between misutilization by patient-visit (binary) and ICD class.

	Coef	SE	p
(Intercept) [1]	−2.7267	0.0342	<0.0001
Secondary ICD Class	1.4677	0.0365	<0.0001

[1] Reference category: Principal ICD code of C61

Table S7. Odds ratios, corresponding 95% CIs, and p-values for the association between misutilization and ICD class.

	OR	95% CI	p
Secondary ICD Class	4.3391	4.0419–4.6634	<0.0001

Table S8. Logistic regression results for the association between misutilization and age groups.

	Coef	SE	p
(Intercept) [1]	-2.1335	0.2493	<0.0001
46-50	-0.6127	0.3028	0.0430
51-55	-0.4331	0.2641	0.1011
56-60	-0.2102	0.2549	0.4096
61-65	0.0109	0.2523	0.9654
66-70	0.4083	0.2512	0.1040
71-75	0.5758	0.2510	0.0218
76-80	0.8485	0.2509	0.0007
81-85	0.9762	0.2511	<0.0001

[1] Reference category: Ages 45 and under

Table S9. Odds ratios, corresponding 95% CIs, and p-values for the association between misutilization and age groups.

	OR	95% CI	p
46-50	0.5419	0.3031-1.0004	0.0430
51-55	0.6485	0.3961-1.1222	0.1011
56-60	0.8104	0.5053-1.3805	0.4096
61-65	1.0110	0.6341-1.7145	0.9654
66-70	1.5043	0.9457-2.5462	0.1040
71-75	1.7785	1.1186-3.0094	0.0218
76-80	2.3362	1.4696-3.9524	0.0007
81-85	2.6543	1.6691-4.4920	<0.0001

Table S10. Logistic regression results for the associations between over- and under-utilization with payor type.

	Under-Utilization			Over-Utilization		
	Coef	SE	p	Coef	SE	p
(Intercept) [1]	-1.7126	0.0187	<0.0001	-2.6699	0.0273	<0.0001
Charity or Indigent	0.2171	0.3074	0.4801	0.0897	0.4646	0.8470
Commercial Indemnity	-1.3405	0.0945	<0.0001	-0.5905	0.1055	<0.0001
Direct Employer Contract	-1.7214	0.5869	0.0034	-0.2315	0.4601	0.6149
Managed Care Capitated	-1.4229	0.3865	0.0002	-0.6259	0.4166	0.1330
Managed Care Non Capitated	-1.5928	0.0625	<0.0001	-0.8475	0.0714	<0.0001
Medicaid Managed Care Capitated	-0.3797	0.1977	0.0548	-0.2125	0.2760	0.4413
Medicaid Managed Care Non Capitated	-0.6935	0.1061	<0.0001	-0.3549	0.1395	0.0110
Medicaid Traditional	0.0485	0.1038	0.6403	-0.1312	0.1632	0.4212
Medicare Managed Care Capitated	-0.3935	0.0584	<0.0001	-0.2000	0.0812	0.0137
Medicare Managed Care Non Capitated	0.0251	0.0338	0.4566	-0.0595	0.0506	0.2389
Other	-1.6997	0.1965	<0.0001	-0.5993	0.1850	0.0012
Other Government Payers	-0.4110	0.0936	<0.0001	-0.1970	0.1285	0.1254
Self-Pay	0.0332	0.1325	0.8022	-0.4824	0.2422	0.0464
Workers Compensation	-1.2831	0.5919	0.0302	-0.0213	0.5174	0.9671

[1] Reference category: Medicare Traditional

Table S11. Odds ratios, corresponding 95% CIs, and p-values for the associations between over- and under-utilization with payor type.

	Under-Utilization			Over-Utilization		
	OR	95% CI	p	OR	95% CI	p
Charity or Indigent	1.2425	0.6511-2.1954	0.4801	1.0938	0.3825-2.4589	0.8470
Commercial Indemnity	0.2617	0.2164-0.3135	<0.0001	0.5540	0.4479-0.6776	<0.0001
Direct Employer Contract	0.1788	0.0439-0.4760	0.0034	0.7933	0.2792-1.7627	0.6149
Managed Care Capitated	0.2410	0.1022-0.4761	0.0002	0.5348	0.2099-1.1066	0.1330
Managed Care Non Capitated	0.2034	0.1795-0.2295	<0.0001	0.4285	0.3717-0.4918	<0.0001

Medicaid Managed Care Capitated	0.6841	0.4548-0.9900	0.0548	0.8086	0.4490-1.3357	0.4413
Medicaid Managed Care Non Capitated	0.4998	0.4036-0.6120	<0.0001	0.7013	0.5276-0.9127	0.0110
Medicaid Traditional	1.0497	0.8525-1.2812	0.6403	0.8770	0.6274-1.1913	0.4212
Medicare Managed Care Capitated	0.6747	0.6009-0.7556	<0.0001	0.8187	0.6962-0.9572	0.0137
Medicare Managed Care Non Capitated	1.0254	0.9596-1.0954	0.4566	0.9422	0.8528-1.0397	0.2389
Other	0.1827	0.1213-0.2629	<0.0001	0.5492	0.3743-0.7750	0.0012
Other Government Payers	0.6630	0.5495-0.7934	<0.0001	0.8212	0.6326-1.0478	0.1254
Self-Pay	1.0337	0.7912-1.3308	0.8022	0.6173	0.3702-0.9621	0.0464
Workers Compensation	0.2772	0.0676-0.7482	0.0302	0.9789	0.2966-2.3816	0.9671

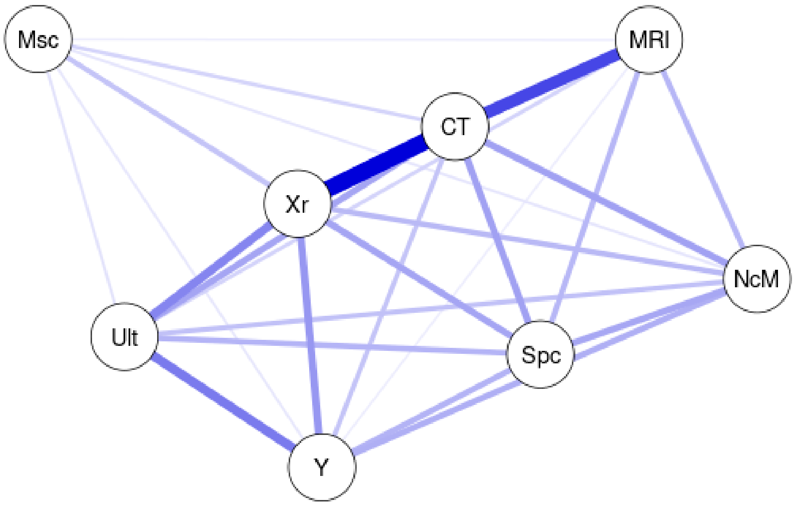

Figure S1. Network analysis for associations (using tetrachoric correlations) among all competing resources and the response resource.

Y=Response Resource (CT scan of pelvis/abdomen without contrast); Xr=X-rays; Ult=Ultrasound; Spc=Special Imaging Techniques; NcM=Nuclear Medicine; CT=CT scan; MRI=MRI & MRA; Msc=Miscellaneous

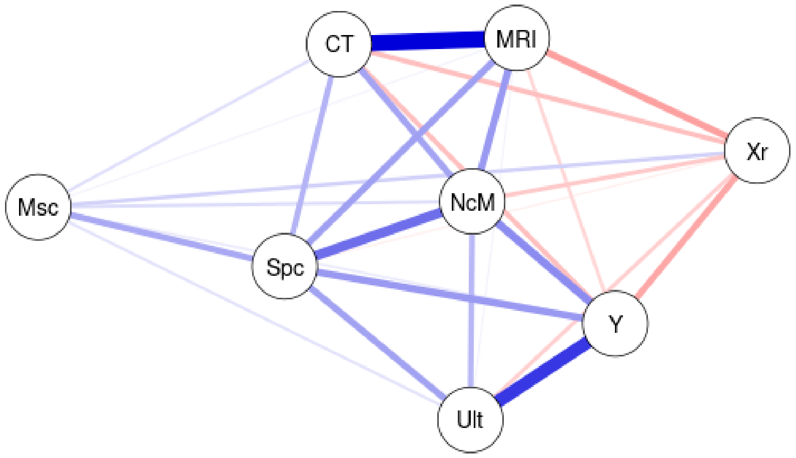

Figure S2. Network analysis for associations (using tetrachoric correlations) of resources for patient-visits with the utilization of at least one competing resource.

Y=Response Resource (CT scan of pelvis/abdomen without contrast); Xr=X-rays; Ult=Ultrasound; Spc=Special Imaging Techniques; NcM=Nuclear Medicine; CT=CT scan; MRI=MRI & MRA; Msc=Miscellaneous

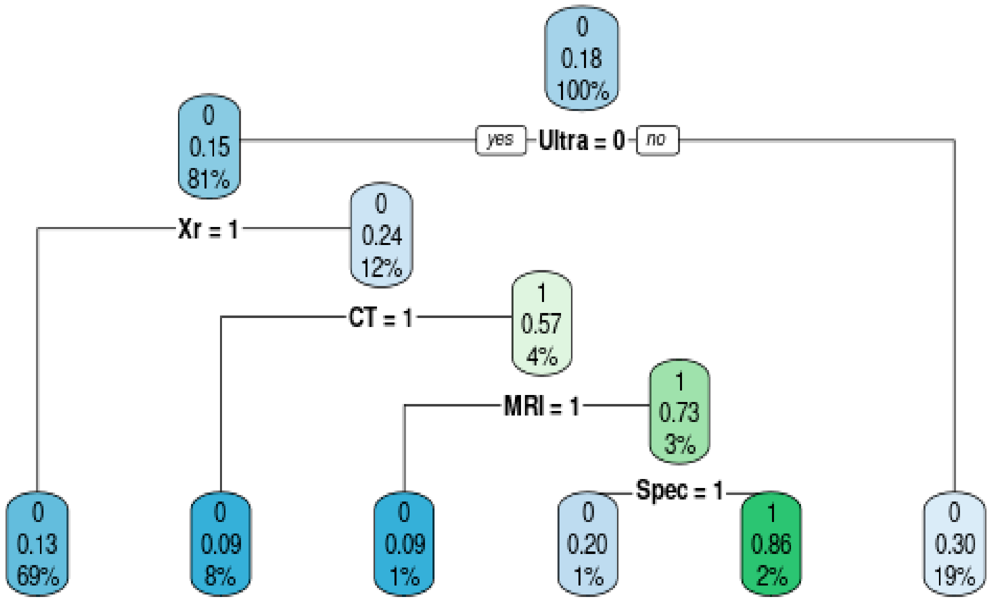

Figure S3. Decision tree analysis to isolate different patterns in resource utilization by demonstrating the value of information of competing resources with the response resource.
